# Magnetic Thermal Ablation Combined with Temporally Coordinated CpG Immunotherapy Promotes cDC1‐Dependent Immune Priming in Hepatocellular Carcinoma

**DOI:** 10.1002/advs.78224

**Published:** 2026-10-22

**Authors:** Jiakun Yang, Nailin Yang, Shuangshuang Ji, Siping Meng, Di Wang, Wenjun Liang, Xuming Bai, Yong Jin, Xingwei Sun

**Affiliations:** ^1^ Department of Interventional Radiology The Second Affiliated Hospital of Soochow University Suzhou People's Republic of China; ^2^ Chemical Engineering and Materials Science Soochow University Suzhou People's Republic of China; ^3^ Institute of Functional Nano & Soft Materials (FUNSOM) Jiangsu Key Laboratory for Carbon‐Based Functional Materials and Devices Soochow University Suzhou People's Republic of China; ^4^ Department of Anesthesiology The Second Affiliated Hospital of Soochow University Suzhou People's Republic of China

**Keywords:** cDC1‐dependent immune priming, CpG oligodeoxynucleotides, hepatocellular carcinoma, polylactic acid/iron oxide magnetic microspheres, transarterial magnetic thermal ablation

## Abstract

Hepatocellular carcinoma (HCC) imposes a major global health burden, with current ablation techniques limited by invasiveness and post‐treatment recurrence. Here, size‐controllable, polylactic acid/iron oxide magnetic microspheres containing 30% Fe_3_O_4_ (P@Fe30 MSs) were fabricated via shirasu porous glass membrane emulsification for transarterial magnetic thermal ablation (MTA). The optimized microspheres exhibit superior magnetothermal performance under an alternating magnetic field, effectively killing tumor cells and triggering immunogenic cell death (ICD). Mechanistically, MTA‐induced tumor disruption cooperates with the TLR9 agonist CpG ODN to accelerate dendritic cell maturation and promote a tumor–TDLN immune‐priming cascade, featuring CD103^+^ cDC1 enrichment and CCR7‐associated cDC1 migration dynamics, functional CD8^+^ T‐cell activation, and systemic memory‐associated immune remodeling. Crucially, direct evaluation of CpG timelines reveals that early post‐MTA CpG administration significantly outperforms delayed delivery, validating a transient post‐ablation immune‐priming window. Furthermore, experiments in Batf3^−^/^−^ mice confirm that this systemic antitumor immunity strictly depends on a cDC1‐ mediated priming pathway. Finally, preclinical validation in an orthotopic rabbit VX2 liver tumor model demonstrates excellent transarterial feasibility, localized metabolic tumor suppression via ^18^F‐FDG PET imaging, and a favorable safety profile. This work establishes a remotely activated transarterial ablation platform that coordinates with temporal innate immunotherapy to drive systemic cDC1‐dependent antitumor responses.

## Graphical abstract

Novel PLA@Fe_3_O_4_ magnetic microspheres were developed for transarterial magnetic hyperthermia ablation (MTA) of hepatocellular carcinoma. While destroying local tumors and inducing immunogenic cell death (ICD), MTA combined with early CpG adjuvant administration significantly promotes cDC1‐dependent immune priming. This synergistic mechanism effectively activates CD8^+^ T cells, triggering robust systemic anti‐tumor immunity to inhibit distant tumors while safely eliminating the primary lesion.

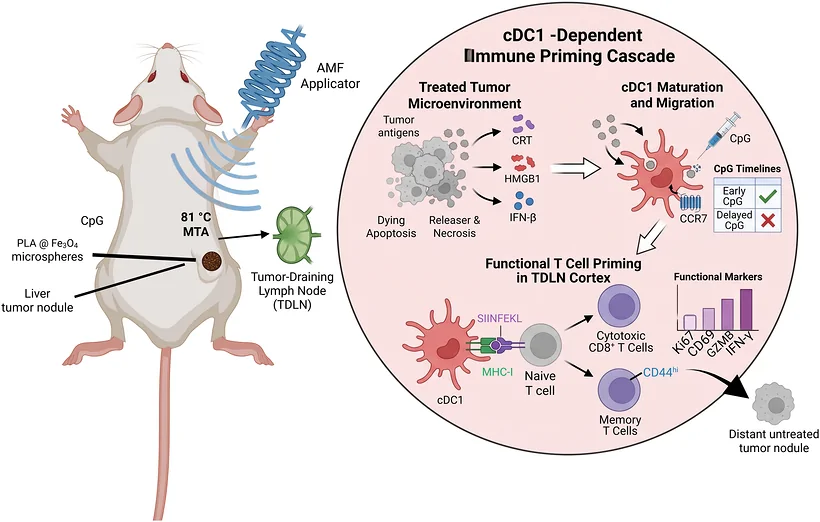

## 1 Introduction

Hepatocellular carcinoma (HCC) is among the leading causes of cancer‐related deaths worldwide. According to the latest statistics from the World Health Organization, primary liver cancer accounts for more than 800,000 deaths each year, with HCC representing the predominant histological subtype [1, 2, 3]. Innovations in clinical treatment strategies have attracted substantial attention. Image‐guided percutaneous puncture ablation technology, with its precise and minimally invasive characteristics, has become one of the preferred treatment options for early‐stage liver cancer [4, 5]. Although good therapeutic effects have been achieved, image‐guided percutaneous puncture ablation still faces the problems of tissue puncture injury, incomplete ablation in anatomically challenging lesions, insufficient systemic antitumor immune activation, and postoperative recurrence [6, 7, 8]. Therefore, there remains a need for alternative local ablation strategies that can achieve effective tumor destruction while enabling systemic immune activation.

Magnetic hyperthermia therapy (MHT) is a remotely activated therapeutic approach in which alternating magnetic fields (AMFs) induce heat generation in magnetic materials, mainly through Néel and Brownian relaxation mechanisms. By elevating the temperature of local tissues, this technology achieves the objective of eradicating diseased cells. MHT is not limited by tissue depth and can achieve non‐contact thermal activation of deeply located tumors [9, 10, 11, 12, 13]. Nanoscale magnetothermal particles (such as Fe_3_O_4_ NPs) were the primary media used in early MHT studies, and their biocompatibility and biomedical potential have been widely investigated [14, 15, 16, 17, 18]. Fe_3_O_4_ NPs are enriched after intravenous injection or intratumoral injection. Under the action of an AMF, local tumor warming can be achieved. However, the limited aggregation of magnetothermal media is restrictive, resulting in a poor warming effect. Moreover, the entry of nanomedia into venous circulation poses safety hazards, limiting its clinical translation [19, 20, 21, 22, 23, 24].

Building on these findings, we propose a strategy for the delivery of magnetothermal media via the arteries: based on the characteristics of polylactic acid (PLA), which has excellent biocompatibility and is suitable for large‐scale production, micron‐sized magnetic iron oxide microspheres were constructed, and transarterial embolization technology was applied to achieve precise enrichment of the microspheres at the tumor site. This procedure ensures that the micrometer‐level embolic medium is precisely enriched in the tumor tissue, thereby enhancing safety. The efficacy of magnetic field‐induced heating can be markedly increased by achieving high accumulation of magnetic thermal media within the tumor. This strategy effectively circumvents the limitations associated with percutaneous puncture, paving the way for a non‐percutaneous, transarterial magnetic thermal ablation strategy: magnetic thermal ablation (MTA).

Immunotherapy has significantly reshaped the landscape of tumor treatment and has demonstrated remarkable clinical benefits in various malignant tumors [25, 26, 27, 28, 29, 30]. However, the clinical response rate of patients with HCC to immunotherapy remains insufficient, generally ranging from 20% to 30% [31, 32, 33]. This therapeutic limitation is attributed mainly to the complex immunosuppressive mechanism within the tumor microenvironment (TME) and defects in tumor antigen presentation [34, 35]. Notably, thermal ablation therapy can not only directly induce tumor cell death, but it can also release tumor antigens and form in situ tumor vaccines while killing tumors [34, 35, 36]. For example, Keisari Y et al. (2023) reported that thermal ablation can promote the release of tumor‐associated antigens and increase the infiltration of immune cells into tumor foci [37]. Therefore, MTA is expected to exert a synergistic effect with immunotherapy. Through rational coordination with innate immune adjuvants, local ablation may be converted into systemic antitumor immune activation.

CpG oligodeoxynucleotides (CpG ODNs) are potent synthetic immune agonists that trigger innate immune responses by activating the Toll‐like receptor 9 (TLR9) pathway [38, 39]. In antitumor immunity, the most characteristic role of CpG ODNs is to significantly increase the maturation and activation of dendritic cells (DCs), thereby promoting the initiation and expansion of CD8^+^ T‐cell priming [40, 41]. This process is accompanied by the secretion of key proinflammatory cytokines (such as TNF‐α and IFN‐γ), forming a positive immune regulatory circuit that synergistically amplifies the antitumor immune response [42, 43, 44]. Notably, DCs in the TME of HCC are often in a state of functional inhibition [45]. Studies have shown that local delivery of CpG ODNs within tumors can effectively reverse the immunosuppressive phenotype of DCs, reshape the TME, and activate the innate immune pathway [46, 47]. While our previous work demonstrated the potential of combining MTA with immune checkpoint blockade (e.g., anti‐PD‐1) to reinvigorate exhausted T cells [48], such strategies primarily function at the late effector phase of immunity (“releasing the brakes”) and rely heavily on pre‐existing T cell infiltration. However, for “cold” tumors lacking initial T cell infiltration, overcoming the upstream defect in antigen cross‐presentation is paramount. Thus, distinct from checkpoint blockade, targeting the earliest phase of innate immune priming (“stepping on the gas”) represents a critical unmet need to generate systemic adaptive immunity from the ground up.

In this study, size‐controllable PLA@Fe_3_O_4_ magnetic microspheres were fabricated using shirasu porous glass membrane emulsification, and the optimized formulation containing 30% Fe_3_O_4_ (P@Fe30 MSs) was selected on the basis of its magnetothermal performance, stability, and biocompatibility. We investigated whether P@Fe30 MS‐mediated MTA could induce tumor‐cell death and immunogenic cell death (ICD)‐associated danger signaling, and whether temporally coordinated CpG administration could enhance the conversion of local ablation into systemic antitumor immunity. Using murine bilateral tumor models, we evaluated distant untreated tumor suppression, compared early versus delayed CpG administration after MTA, and characterized immune responses across treated tumors, TDLNs, and spleens. We further assessed the requirement for cDC1s using Batf3^−^/^−^ mice. Finally, the transarterial feasibility, local tumor control, and safety of P@Fe30 MS‐mediated MTA were evaluated in an orthotopic rabbit VX2 liver tumor model. Together, this work defines a transarterial MTA platform that can be integrated with temporally coordinated CpG immunotherapy to promote cDC1‐dependent immune priming and systemic antitumor responses in HCC, as schematically illustrated in Scheme 1.

**SCHEME 1:**
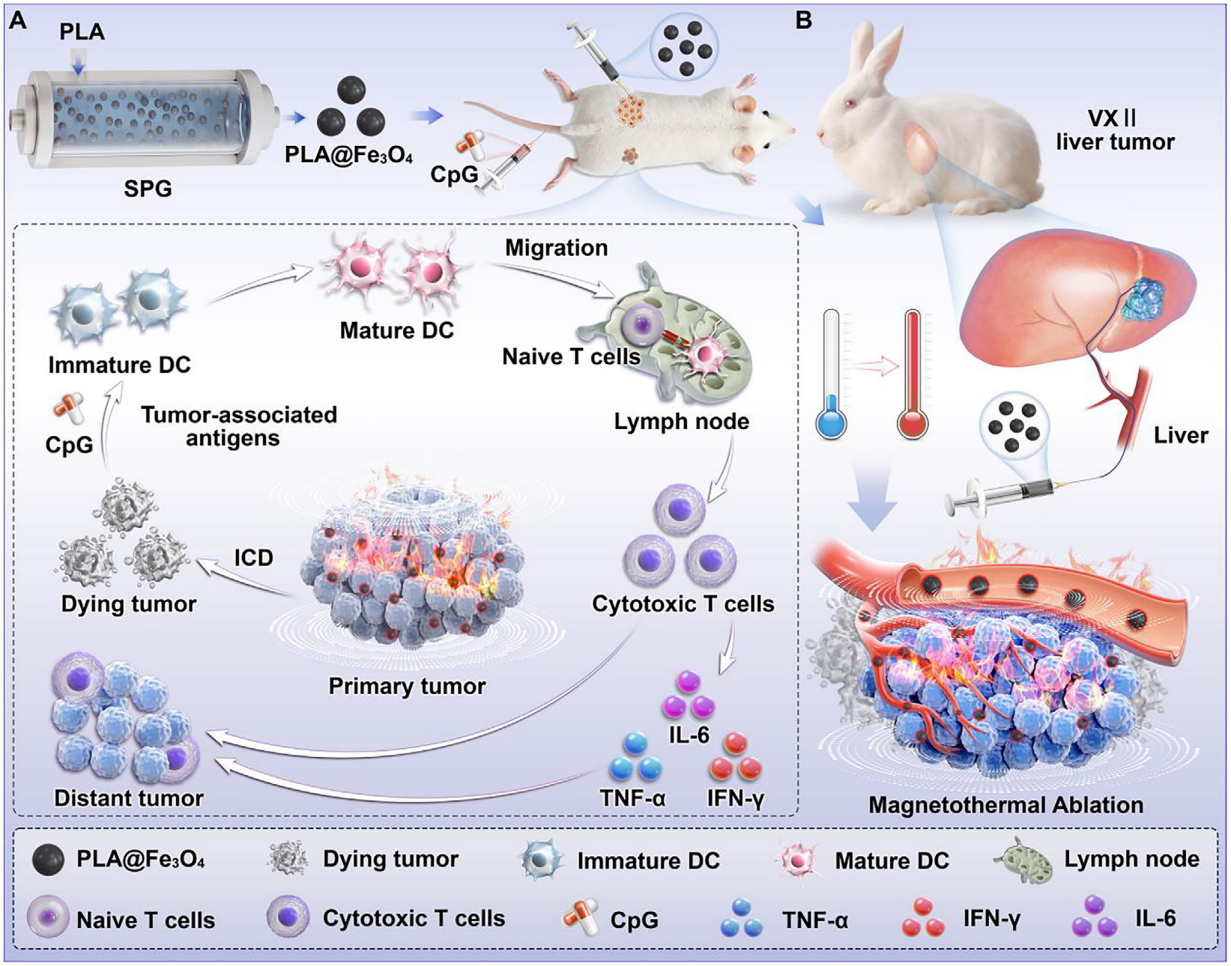
Schematic illustration of PLA@Fe_3_O_4_ microsphere‐mediated magnetic thermal ablation combined with temporally coordinated CpG immunotherapy for HCC. (a) PLA@Fe_3_O_4_ microspheres were fabricated by SPG membrane emulsification and applied to MTA combined with CpG immunotherapy in a murine bilateral HCC tumor model. MTA‐induced tumor destruction and danger‐signal release were coordinated with CpG administration to enhance DC activation and systemic antitumor responses. (b) The feasibility, therapeutic efficacy, and safety of transarterial PLA@Fe_3_O_4_ MS‐mediated MTA were evaluated in an orthotopic rabbit VX2 liver tumor model.

## 2 Results

### 2.1 Construction and Characterization of the PLA@Fe_3_O_4_ MSs

Owing to their small size, nanoscale media may pose a safety hazard in clinical practice. The diameters of commonly used embolic microspheres range from 50 µm to 700 µm. This size range not only effectively prevents venous escape but also enables precise enrichment of microspheres in tumor‐supplying arteries [49]. On this basis, PLA MSs and polylactic acid/iron oxide magnetic microspheres with different Fe_3_O_4_ contents were fabricated using the SPG technique. The magnetic microspheres were denoted as P@FeX MSs, where X represents the Fe_3_O_4_ content of 10%, 20%, or 30%. The SPG membrane emulsification process enables scalable preparation of microdroplets with precise particle size control and excellent monodispersity [50]. Monodisperse PLA MSs, P@Fe10 MSs, P@Fe20 MSs, and P@Fe30 MSs were successfully prepared (Figure 1A). To quantify the monodispersity, particle size analysis was performed using ImageJ software. The pure PLA microspheres exhibited a mean diameter of 48.3 ± 2.7 µm with a Polydispersity Index (PDI) of 0.011. With the increase of Fe_3_O_4_ loading to 30% (P@Fe30), the mean diameter slightly increased to 51.7 ± 3.6 µm (PDI = 0.022), likely due to the increased viscosity of the dispersed phase. Notably, the PDI for all groups remained below 0.05, confirming the high precision of the SPG technique. During formulation optimization, regular and monodisperse PLA@Fe_3_O_4_ microspheres could be obtained at Fe_3_O_4_ fractions up to 30%. Further increasing the Fe_3_O_4_ fraction under the same SPG processing conditions resulted in markedly less uniform particle formation, with irregular, deformed, and fragmented structures observed (Figure S1). Therefore, P@Fe30 was selected as the highest practically feasible formulation that preserved the spherical morphology and size uniformity required for subsequent transarterial application. Optical microscopy revealed that the PLA and P@Fe30 MSs had regular spherical shapes (Figure 1B). SEM revealed that PLA microspheres loaded with nanoiron oxide presented a micrometer‐scale spherical shape (Figure 1C). Thermogravimetric analysis (TGA) confirmed the Fe_3_O_4_ loading capacity of the synthesized microspheres (Figure 1D). Elemental analysis indicated that the P@Fe30 MSs contained three elements, C, O, and Fe, further verifying the successful preparation of P@Fe30 MSs (Figure 1E).

**FIGURE 1:**
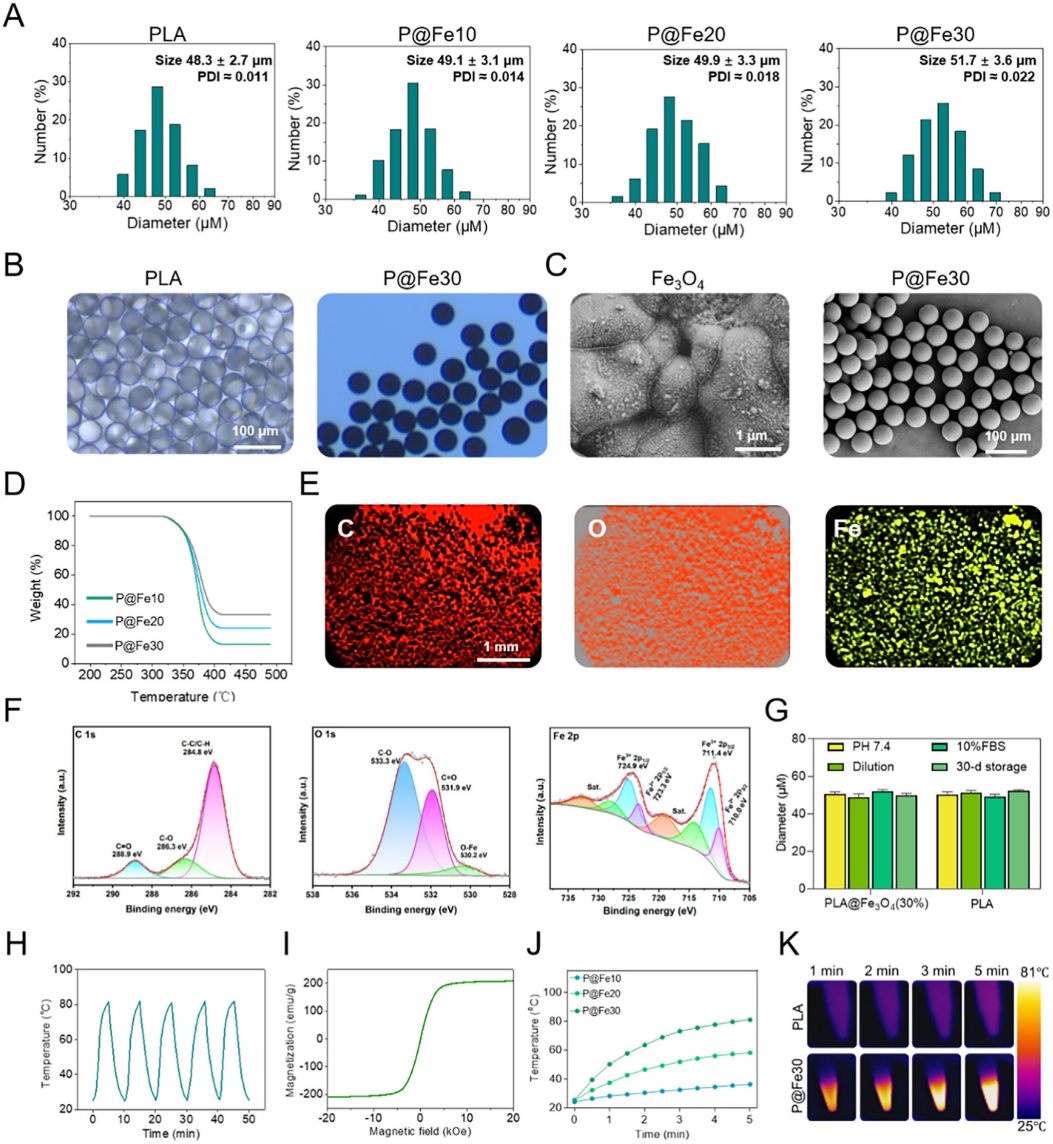
Characterization of PLA@Fe_3_O_4_ MSs. (A) Size distribution of PLA MSs and P@FeX MSs with different Fe_3_O_4_ contents (mean ± s.d., *n* = 200, calculated via ImageJ). (B) Bright‐field images of PLA and P@Fe30 MSs; scale bar = 100 µm. (C) SEM images of Fe_3_O_4_ nanoparticles and P@Fe30 MSs. (D) TGA curves of P@FeX MSs with varying Fe_3_O_4_ contents. (E) EDS spectrum and (F) XPS spectrum of P@Fe30 MSs. (G) Structural stability of PLA and P@Fe30 MSs during the 30‐day observation period (mean ± s.d., *n* = 3 independent samples). (H) Cyclic heating curve of P@Fe30 MSs. (I) Hysteresis loop of P@Fe30 MSs. (J) Heating profiles of P@FeX MSs with varying Fe_3_O_4_ contents upon 5 min of AMF exposure. (K) Infrared thermal imaging of PLA MSs and P@Fe30 MSs.

To further determine the composition of the material, an X‐ray photoelectron spectroscopy (XPS) analysis was conducted. The survey photoelectron spectrum confirmed the presence of C 1s, O 1s, and Fe 2p, as displayed in Figure 1F. The fine spectrum of C 1s could be divided into three peaks at 284.8 eV, 286.3 eV, and 288.9 eV, which were derived from C─C/C─H, C─O, and C═O, respectively, in PLA. XPS analysis of O 1s indicated that 530.2 eV corresponded to the Fe─O metal bond, and the peaks at 531.9 and 533.3 eV were attributed to C═O and C–O in PLA, respectively. XPS analysis of Fe 2p indicated that the peaks were located at 711.4 eV and 724.9 eV, corresponding to Fe^3+^ 2p_3/2_ and Fe^3+^ 2p_1/2_, respectively. Moreover, the ratio of Fe^2+^ to Fe^3+^ was approximately 1:2, corresponding to Fe_3_O_4_. XPS results verified the successful loading of Fe_3_O_4_ onto the PLA microspheres. Additionally, both PLA and P@Fe30 MSs maintained their structural integrity during the 30‐day stability assessment in PBS supplemented with 10% FBS (Figure 1G). Furthermore, their particle size was retained even after a 100‐fold dilution with PBS (Figure 1G). To further evaluate the stability of the microspheres under physiological conditions relevant to embolization, P@Fe30 MSs were incubated in fresh rabbit plasma for 48 h. As shown in Supplementary Figure S2, the microspheres demonstrated excellent size stability, confirming their structural integrity within a blood‐mimicking environment. Unloaded PLA microspheres showed gradual degradation in physiological saline at 37°C, with the residual mass decreasing to 68.67% ± 4.93% by Day 60 (Figure S3A). Optical microscopy further revealed progressive morphological changes during incubation (Figure S3B). ICP‐MS analysis further showed limited iron release from P@Fe30 microspheres during 30 days of incubation in physiological saline at 37°C, with a cumulative release of 4.47 ± 0.55% at Day 30 (Figure S4). To evaluate the key physicochemical properties and magnetothermal performance of the magnetic microspheres, P@Fe30 MSs with a Fe_3_O_4_ ratio of 30% were placed in a magnetic field. During a continuous 5‐min heating cycle, the temperature reached 81°C (Figure 1H), and stable magnetothermal efficiency was maintained. Furthermore, the hysteresis loop indicated that the saturation magnetization of P@Fe30 was 204 emu/g (Figure 1I). P@FeX MSs with different Fe_3_O_4_ ratios were investigated for their heating performance in an AMF (frequency of 500 kHz and magnetic field strength of 5.0 kA/m) (Figure 1J). Under a constant AMF intensity, the heating efficacy of the P@FeX MSs, as measured by the temperature increase, was predominantly governed by their Fe_3_O_4_ content. The enhanced heating performance with increasing Fe_3_O_4_ content is attributable to the greater amount of magnetically responsive material available for energy dissipation under the AMF. Because the relative contributions of different magnetic‐loss mechanisms were not specifically resolved in this study, no single microscopic heating mechanism is assigned here. Finally, the heating capacity of the P@Fe30 MSs after 5 min of AMF exposure was further confirmed by thermal imaging (Figure 1K).

### 2.2 In Vitro MTA

First, we investigated the biocompatibility of P@Fe30 MSs using L929 fibroblasts, H22 mouse hepatoma cells, and VX2 rabbit hepatoma cells. An MTT cytotoxicity assay verified that P@Fe30 did not significantly affect any of the above three types of cells (Figure 2A), indicating its excellent biocompatibility. Hemolysis tests indicated that P@Fe30 MSs had good blood compatibility (Figure 2B), providing further strong support for the safety of transarterial embolization.

**FIGURE 2:**
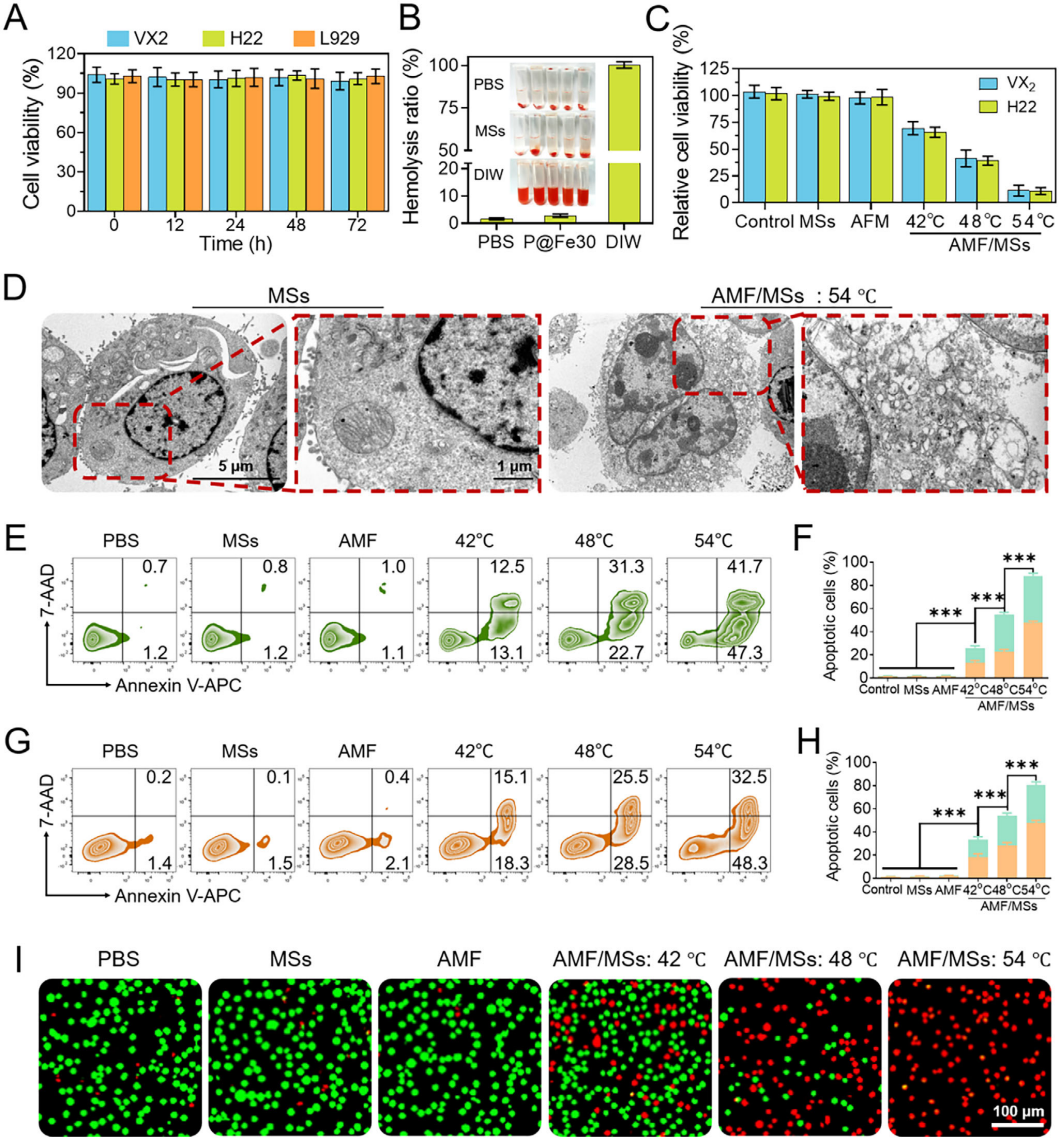
In vitro Magnetic thermal ablation (MTA) Efficacy. (A) Relative cell viability of L929, VX2, and H22 cells incubated with P@Fe30 MSs for 48 h (mean ± s.d., *n* = 5). (B) Hemolysis assay of P@Fe30 MSs (mean ± s.d., *n* = 3). (C) Relative survival rates of H22 and VX2 cells after MTA at different temperatures (mean ± s.d., *n* = 5). (D) TEM images showing H22 cell morphology post‐MTA. Scale bars = 5 µm and 1 µm for the low‐ and high‐magnification images, respectively. (E–H) Representative flow cytometry plots and quantitative analysis of apoptosis in H22 (E, F) and VX2 (G, H) cells (mean ± s.d., *n* = 4). (I) Representative confocal fluorescence images of Calcein AM (live, green)/PI (dead, red) stained H22 cells post‐MTA (scale bar = 100 µm). Statistical significance was calculated using one‐way ANOVA with Tukey's multiple comparisons test. ^*^ *p* < 0.05, ^**^ *p* < 0.01, ^***^ *p* < 0.001.

To test the ability of P@Fe30 MSs to kill tumor cells in vitro, H22 and VX2 cells were exposed to an AMF together with P@Fe30 MSs (D = 50 µm, Fe_3_O_4_ content of 30%) for 5 min, with the temperature controlled at 42°C, 48°C and 54°C. Exposure at 48°C led to the destruction of ∼60% of tumor cells, whereas a higher temperature of 54°C resulted in a more substantial cell death rate of ∼90% (Figure 2C). Bioelectron microscopy revealed that the magnetic thermal effect of the AMF/MSs caused significant apoptosis of H22 cells (Figure 2D). The cell membrane structure of the tumor cells in the magnetic thermal group was significantly damaged, whereas that in the control group remained intact, indicating that the thermal stimulation induced by the AMF/MSs could effectively kill the tumor cells.

Furthermore, magnetothermal treatment with AMF/MSs had a significant pro‐apoptotic effect on H22 cells, as evidenced by flow cytometry (Figure 2E). AMF/MSs induced apoptosis in approximately 89.0% of H22 cells at 54°C, which was significantly different from the apoptosis rates at 42°C (25.6%) and 48°C (54.0%) (Figure 2F); by contrast, almost no apoptosis occurred in the groups treated with MSs or AMF alone. In VX2 cells, the magnetic thermal effect of P@Fe30 MSs was similar to the apoptotic effect (Figure 2G,H). To exclude potential pro‐apoptotic effects of either component alone, additional controls comprising PBS, MS‐only, and three AMF‐only conditions were evaluated (Figure S5). The three AMF‐only groups were exposed to the respective field strengths used for the AMF/MS treatments, reaching 42°C, 48°C, and 54°C. No significant difference in apoptosis was detected among these five control groups, indicating that neither the microspheres alone nor AMF exposure across the tested field strengths appreciably induced H22 cell apoptosis.

Calcein AM/PI co‐staining revealed minimal red fluorescence (indicating cell death) in the control groups treated with AMF or MSs alone (Figure 2I). Conversely, the AMF/MSs group exhibited a strong temperature‐dependent cytotoxic effect. We additionally evaluated the cellular response to high‐temperature MTA reaching approximately 81°C. Calcein AM/PI staining revealed extensive loss of viable H22 cells and widespread PI‐positive staining (Figure S6), demonstrating profound tumor‐cell destruction under this high‐temperature ablative condition. Notably, this high‐temperature experiment was designed to assess the cellular consequence of the ablative condition reached during MTA, whereas the controlled 42–54°C experiments were used to characterize temperature‐dependent apoptosis and ICD‐associated danger signaling.

To further assess whether the MTA strategy can elicit antitumor immune responses by inducing ICD, the cell membrane surface exposure of typical markers of ICD, the proapoptotic protein calreticulin (CRT), and the nuclear release of high mobility group protein B1 (HMGB1) were detected (Figure 3A). Notably, PBS, MS‐only, AMF‐only, and AMF/MS groups were included in the ICD‐associated analyses to distinguish the magnetothermal effects from potential effects of either component alone. Flow cytometry analysis revealed that the surface expression level of CRT in H22 cells in the AMF/MSs treatment group was significantly greater than that in the control group (Figure 3B,C; ^***^ *p* < 0.001). Moreover, ELISA revealed that HMGB1 release was significantly increased after AMF/MSs treatment (Figure 3D; ^***^ *p* < 0.001). Consistently, IFN‐β levels in the culture supernatant were significantly increased following AMF/MSs treatment (Figure S7), further supporting an ICD‐associated inflammatory response induced by MTA. In addition, extracellular ATP levels in the culture supernatant were significantly increased after AMF/MS treatment (Figure 3E; ^***^ *p* < 0.001), further supporting MTA‐induced ICD‐associated danger signaling.

**FIGURE 3:**
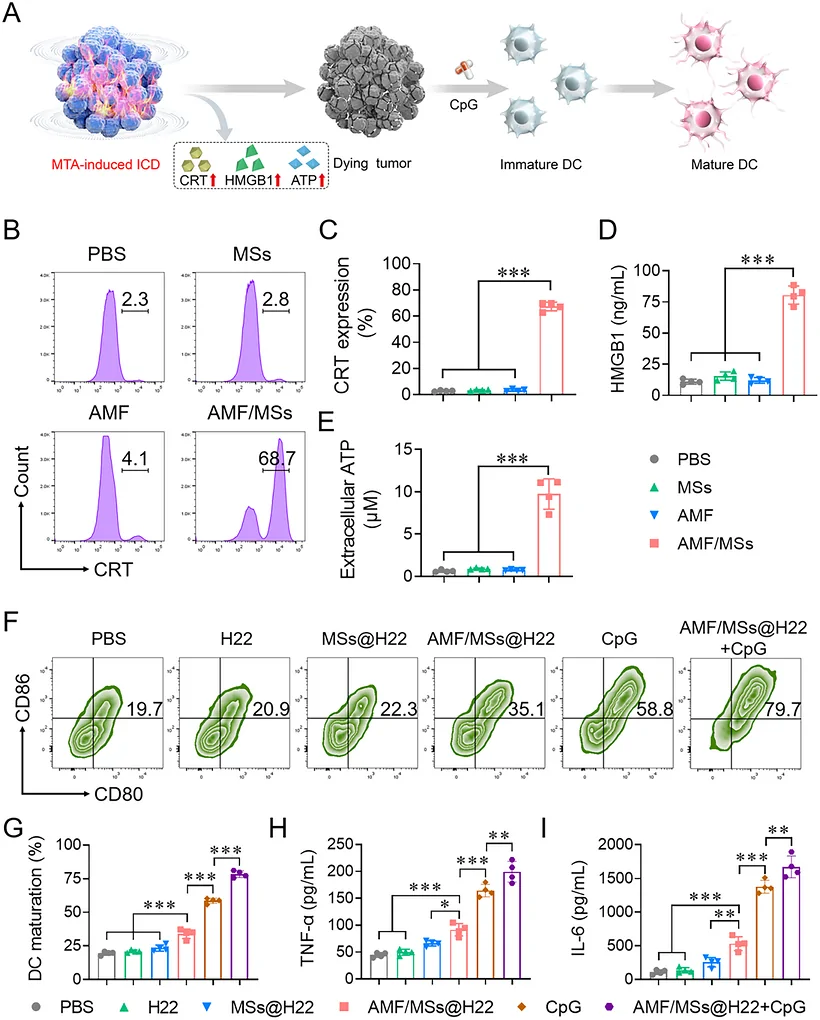
MTA‐induced Immunogenic Cell Death (ICD) and Dendritic Cell Maturation. (A) Schematic illustration of MTA‐induced ICD. (B, C) Representative flow cytometry plots (B) and quantitative analysis (C) of surface Calreticulin (CRT) expression on H22 cells (mean ± s.d., *n* = 4). (D) ELISA quantification of extracellular HMGB1 release (mean ± s.d., *n* = 4). (E) Extracellular ATP levels in the culture supernatants of H22 cells after the indicated treatments (mean ± s.d., *n* = 4). (F, G) Representative flow cytometry plots (F) and quantitative analysis (G) of BMDC maturation (CD80^+^CD86^+^) after co‐culture with treated tumor cells (mean ± s.d., *n* = 4). (H, I) ELISA quantification of TNF‐α (H) and IL‐6 (I) in the co‐culture supernatant (mean ± s.d., *n* = 4). Statistical significance was calculated using one‐way ANOVA with Tukey's multiple comparisons test. ^*^ *p* < 0.05, ^**^ *p* < 0.01, ^***^ *p* < 0.001.

To evaluate the effects of AMF/MS‐mediated tumor‐cell treatment and CpG on BMDC maturation, differentially treated H22 cells were co‐cultured with BMDCs for 24 h in a Transwell system (Figure 3F). BMDCs co‐cultured with AMF/MS‐treated H22 cells showed a modest increase in the proportion of CD80^+^CD86^+^ cells compared with the untreated H22‐cell condition (35.1% vs. 20.9%). In comparison, CpG alone induced a substantially greater increase in BMDC maturation to 58.8%. The combination further increased BMDC maturation to 79.7%, which was significantly higher than that observed with CpG alone (Figure 3G; ^***^ *p* < 0.001). To further assess BMDC antigen cross‐presentation, an OVA model‐antigen assay was performed using B16‐OVA cells and C57BL/6‐derived BMDCs. MTA‐treated B16‐OVA cells increased surface SIINFEKL–H‐2K^b^ presentation on BMDCs, with the highest level observed after MTA + CpG treatment (Figure S8), supporting enhanced antigen‐specific MHC‐I cross‐presentation.Consistently, TNF‐α and IL‐6 levels in the co‐culture supernatant showed a comparatively modest increase in the AMF/MS‐treated H22‐cell condition, a greater increase with CpG, and a further elevation with the combination (Figure 3H,I). CpG alone showed no appreciable effect on H22 cell viability at the 2 µm concentration used in our in vitro experiments, although a modest reduction was observed at substantially higher concentrations (Figure S9). These findings indicate that the AMF/MS‐treated tumor‐cell condition provided additional immunostimulatory signals that augmented the strong BMDC‐activating effect of CpG.

### 2.3 In Vivo Experiments

To investigate whether MTA could induce systemic antitumor immune responses and whether its combination with CpG could suppress the growth of distant untreated tumors, we established a bilateral H22 tumor model in mice (Figure 4A). Under AMF exposure, the temperature of tumors containing P@Fe30 MSs rapidly increased to approximately 81°C within 5 min, whereas no appreciable temperature increase was observed in the AMF‐only group (Figure 4B,C). The volume of distant tumors in each group was assessed on Day 12 following different treatment measures of the primary tumor (Figure 4D,E). To strictly evaluate the systemic immune response against the distant tumors, the primary tumors in the control and CpG‐only groups were surgically resected to standardize the tumor burden against the ablated groups. The results revealed that distant tumor growth was not inhibited in the control group, whereas tumor growth was significantly inhibited in the AMF/MSs group compared with the control group. Compared with that of the MTA group, the therapeutic effect of the CpG group on distant tumors was significantly improved, likely due to the systemic activation of APCs against existing antigens in the distant metastases. The combined MTA + CpG treatment produced the strongest suppression of distant tumor growth and a survival advantage, with four mice surviving beyond 30 days (Figure 4F). This enhanced outcome was corroborated by H&E staining, which revealed superior tumor suppression compared with all the other groups (Figure 4G), confirming the safety of the treatment in major organs (Figure S10). Immunohistochemical (IHC) TUNEL staining further confirmed (Figure 4H) that the combined treatment group significantly enhanced the apoptosis‐inducing effect on distant tumors, demonstrating stronger antitumor killing efficacy.

**FIGURE 4:**
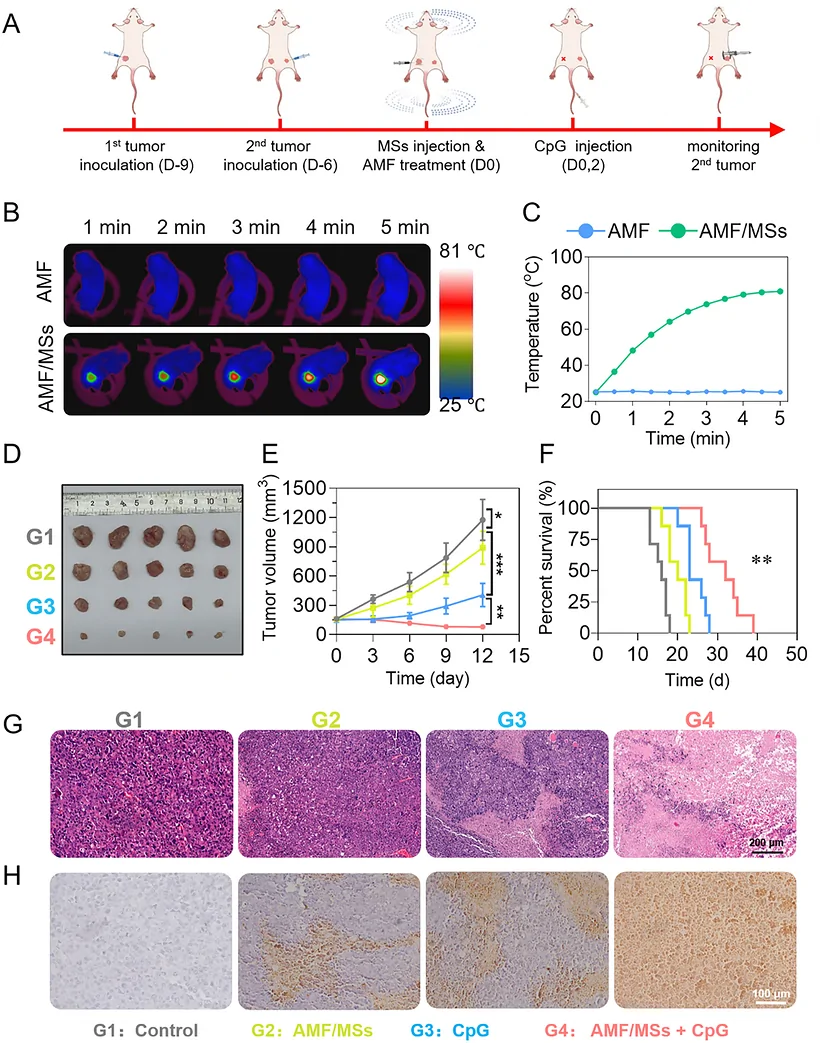
In Vivo enhanced Antitumor Efficacy in a Bilateral Tumor Model. (A) Schematic of the treatment regimen. (B) Representative infrared thermal images and (C) temperature curves during MTA. (D) Digital photographs of excised distal tumors on Day 12 (*n* = 5 mice). (E) Tumor growth curves of distant tumors (mean ± s.d., *n* = 7 mice). (F) Kaplan‐Meier survival curves of mice (*n* = 7 mice). (G) H&E staining and (H) IHC‐TUNEL staining of distal tumor sections (scale bar: 200 µm for H&E, 100 µm for TUNEL). Statistical significance was calculated using one‐way ANOVA for tumor volume and the log‐rank test for survival analysis. ^*^ *p* < 0.05, ^**^ *p* < 0.01, ^***^ *p* < 0.001.

### 2.4 Immunomodulatory Effects of Combinatorial MTA and Immunotherapy

To elucidate whether the combination of MTA and CpG induces systemic immune remodeling at untreated tumor sites, we characterized immune cell populations within distant tumors by flow cytometry on Day 2 after treatment. In this therapeutic cohort, primary tumors in the MTA‐containing groups were completely ablated, whereas primary tumors in the PBS and CpG groups were surgically removed to enable evaluation of distal tumor immune responses after local tumor elimination. The results of the flow cytometry analysis demonstrated that after treatment with the CpG adjuvant, the infiltration percentages of immune cells, such as DCs (Figure 5A,E), CD4^+^ T cells, CD8^+^ T cells (Figure 5B,F), and IFN‐γ^+^CD8^+^ T cells (Figure 5C,G), at the tumor site significantly increased compared with those in the control group and the AMF/MSs group. After combined treatment with AMF/MSs, the proportion of the above‐mentioned immune‐related cells further increased. In contrast to the other groups, the AMF/MSs + CpG combination distinctly suppressed the accumulation of CD4^+^ FoxP3^+^ regulatory T cells (Tregs) in the TME (Figure 5D,H).

**FIGURE 5:**
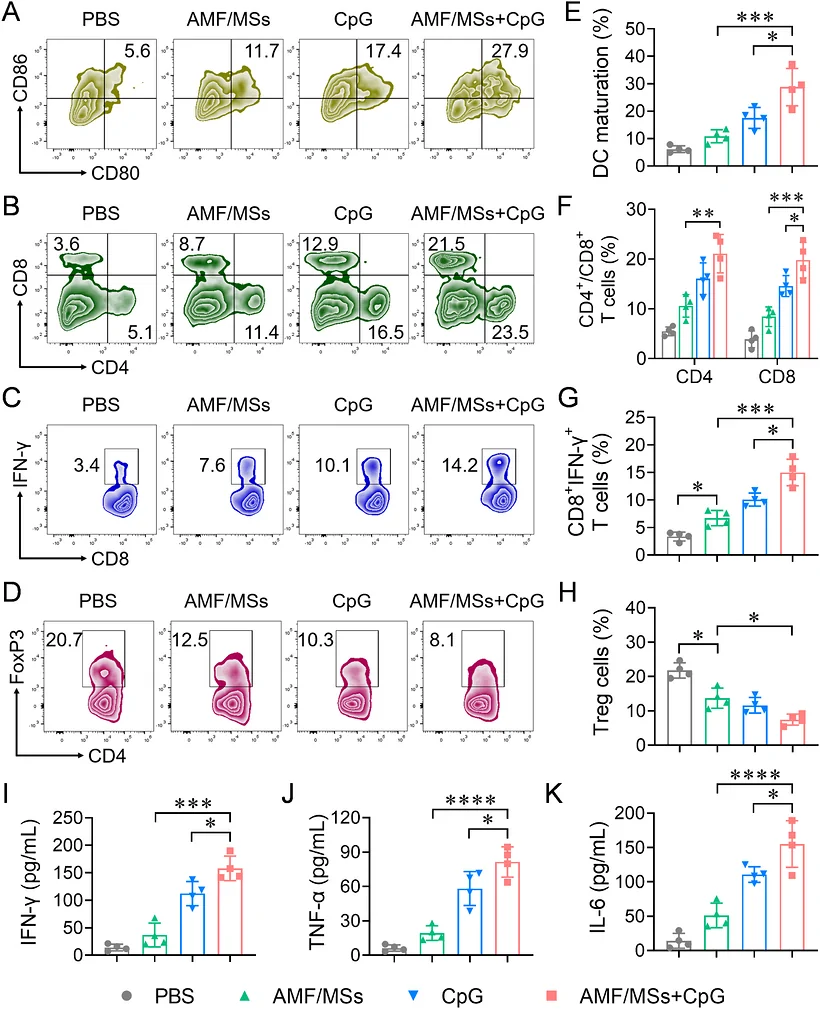
Immune Microenvironment Remodeling in Distant Tumors. (A–D) Representative flow cytometry plots of (A) mature DCs (CD80^+^CD86^+^), (B) CD4^+^ and CD8^+^ T cells, (C) IFN‐γ^+^ CD8^+^ T cells, and (D) Tregs (FoxP3^+^CD4^+^) in distal tumors. (E–H) Quantitative analysis of the corresponding immune cell populations (mean ± s.d., *n* = 4). (I–K) ELISA quantification of intratumoral (I) IFN‐γ, (J) TNF‐α, and (K) IL‐6 levels (mean ± s.d., *n* = 4). Statistical significance was calculated using one‐way ANOVA with Tukey's multiple comparisons test. ^*^ *p* < 0.05, ^**^ *p* < 0.01, ^***^ *p* < 0.001, ^****^ *p* < 0.0001.

To benchmark the CpG‐containing MTA regimen against the previously used checkpoint blockade‐based combination strategy, we introduced anti‐PD‐1‐related treatment groups in the bilateral tumor model (Figure). The treatment groups included PBS, anti‐PD‐1, MTA + anti‐PD‐1, and MTA + anti‐PD‐1 + CpG. Incorporation of CpG into the MTA+anti‐PD‐1 regimen further suppressed distal tumor progression (Figure) and extended survival compared with MTA+anti‐PD‐1 treatment (Figure). Consistently, H&E (Figure) and TUNEL (Figure) staining of distal tumors showed more extensive tumor tissue destruction and increased tumor‐cell death in the MTA+anti‐PD‐1+CpG group. These findings suggest that, although MTA can cooperate with PD‐1 blockade to improve systemic antitumor efficacy, checkpoint inhibition alone may not fully exploit the immunogenic potential generated by MTA‐induced tumor destruction. These results indicate that CpG provides an additional upstream immune‐priming contribution to the MTA‐based regimen, which is complementary to PD‐1 blockade‐mediated effector‐phase regulation.

To determine whether these frequency changes reflected alterations in cellular abundance rather than relative shifts in immune‐cell composition alone, we further quantified total CD45^+^ leukocytes and CD8^+^ T cells normalized to distant‐tumor mass. MTA + CpG significantly increased the absolute abundance of CD8^+^ T cells, whereas total CD45^+^ leukocyte abundance showed only a modest change (Figure S11A,B). These findings support an increase in the absolute abundance of tumor‐infiltrating CD8^+^ T cells and indicate that the observed CD8^+^ T‐cell response cannot be explained solely by treatment‐induced changes in the relative composition of other leukocyte populations.

Finally, ELISA revealed that the release of interferon‐γ (IFN‐γ) (Figure 5I), tumor necrosis factor‐α (TNF‐α) (Figure 5J), and interleukin‐6 (IL‐6) (Figure 5K) further increased after the combined AMF/MSs and CpG treatment. In summary, the combined strategy of AMF/MS‐induced MTA and a CpG adjuvant could reshape the TME by activating antigen‐presenting cells, enhancing effector T‐cell infiltration, inhibiting the immunosuppressive function of Tregs, and promoting the secretion of proinflammatory factors, thereby achieving synergistic amplification of systemic antitumor immune responses. These findings support the ability of the combined regimen to amplify systemic antitumor immune responses beyond the locally treated primary tumor.

To functionally determine whether CD8^+^ T cells contribute to the systemic control of untreated distal tumors, we further performed an in vivo CD8^+^ T‐cell depletion experiment. CD8^+^ T‐cell depletion markedly attenuated the control of untreated distal tumors by MTA + CpG (Figure S14), demonstrating that CD8^+^ T cells make a major functional contribution to the full systemic antitumor efficacy of the combination regimen.

To clarify the temporal rationale for combining CpG with MTA, we first analyzed early immunological changes in the primary tumor microenvironment 24 h after AMF/MSs‐mediated ablation. MTA induced early ICD‐associated changes in the treated tumor, including altered CRT and HMGB1 immunoreactivity together with IFN‐β upregulation (Figure 6A–C), indicating the rapid establishment of a local inflammatory response after ablation. These results suggested the presence of an early post‐ablation immune‐priming window.

**FIGURE 6:**
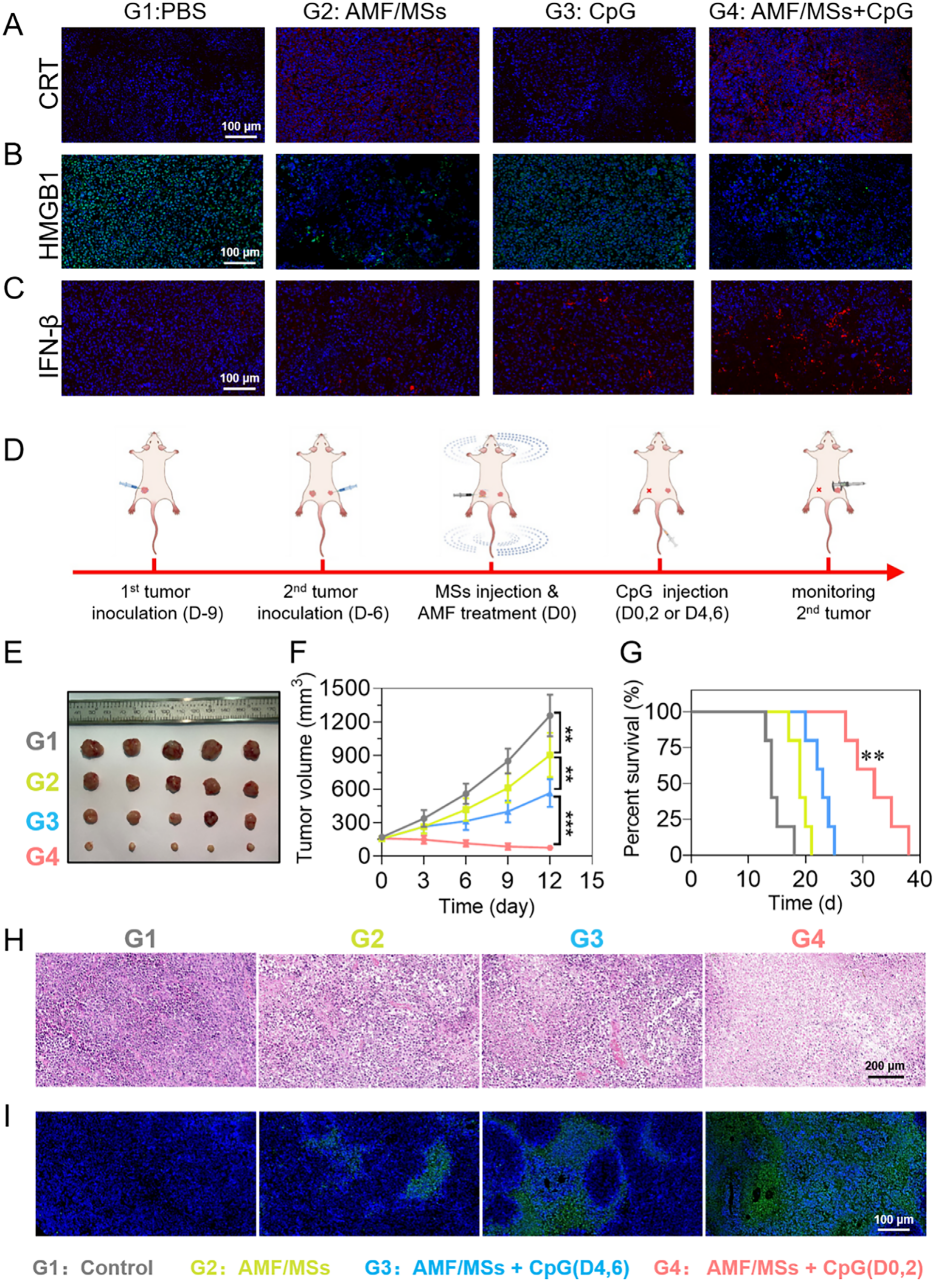
Early post‐MTA immune‐priming window and temporal dependence of CpG efficacy. (A–C) Representative immunofluorescence images of primary tumor sections collected 24 h after AMF/MSs‐mediated ablation, showing changes in CRT (red; A), HMGB1 (green; B) immunoreactivity and IFN‐β upregulation (magenta; C). Nuclei were counterstained with DAPI (blue). Scale bar: 100 µm. These results indicate the rapid establishment of a danger‐signal‐rich inflammatory milieu within 24 h of MTA, defining an early post‐ablation immune‐priming window. (D) Schematic of the bilateral tumor model and CpG timing regimen. Following AMF/MSs‐mediated ablation of the primary tumor, CpG was administered either during the early post‐MTA window (Day 0 and Day 2) or during a delayed window (Day 4 and Day 6). (E) Representative digital photographs of excised distal tumors from each treatment group on Day 12. G1: Control; G2: AMF/MSs; G3: AMF/MSs + CpG (Day 4/6); G4: AMF/MSs + CpG (Day 0/2). (F) Distal tumor growth curves (*n* = 5 mice per group). (G) Kaplan–Meier survival curves (*n* = 5 mice per group). (H) Representative H&E staining of distal tumor sections. Scale bar: 200 µm. (I) Representative TUNEL staining of distal tumor sections. Scale bar: 100 µm. Data are presented as mean ± s.d. Statistical significance was assessed by one‐way ANOVA with Tukey's post hoc test for tumor volume comparisons and by the log‐rank test for survival analysis. ^*^ *p* < 0.05, ^**^ *p* < 0.01, ^***^ *p* < 0.001.

We then directly evaluated whether CpG efficacy depends on its timing after MTA using a bilateral tumor model. CpG was administered either early on Day 0/Day 2 or later on Day 4/Day 6 after MTA (Figure 6D). Compared with delayed CpG administration, early CpG treatment produced smaller excised distal tumors (Figure 6E), stronger distal tumor suppression (Figure 6F), improved survival (Figure 6G), more extensive histological tumor destruction (Figure 6H), and increased TUNEL‐positive tumor cell death (Figure 6I). These findings support temporal coordination of CpG administration with MTA‐induced tumor disruption and ICD‐associated inflammatory signaling to enhance systemic antitumor responses.

Having established that MTA induces an early ICD‐associated inflammatory response and that CpG administration during this early window is therapeutically superior to delayed treatment, we next investigated how MTA‐conditioned local inflammation cooperates with CpG to shape the tumor–TDLN immune‐priming cascade. To this end, we performed flow‐cytometric immune profiling of treated primary tumors, TDLNs, and spleens using an independent bilateral subcutaneous tumor cohort. Unlike the distal‐tumor profiling experiment, which was designed to assess systemic immune remodeling after complete primary‐tumor ablation or removal, this mechanistic cohort used partial ablation in MTA‐containing groups to preserve residual viable and peri‐ablation tumor tissues for local immune analysis.

In both treated primary tumors (Figure 7A,B) and TDLNs (Figure 7C,D), MTA or CpG monotherapy increased the frequency of total DCs, whereas MTA + CpG resulted in the highest DC frequency. Consistently, CD103^+^ cDC1s were enriched in both compartments after treatment, with the highest levels observed in the MTA + CpG group (Figure 7E–H). To complement these frequency‐based measurements, we further quantified the absolute abundance of CD103^+^ cDC1s in treated primary tumors and TDLNs. MTA + CpG significantly increased the absolute abundance of CD103^+^ cDC1s in both treated primary tumors and TDLNs compared with the control group (Figure S11C,D). The concordance between the relative‐frequency and absolute‐abundance measurements supports cDC1 accumulation across the treated tumor–TDLN axis rather than a change in cDC1 representation alone.

**FIGURE 7:**
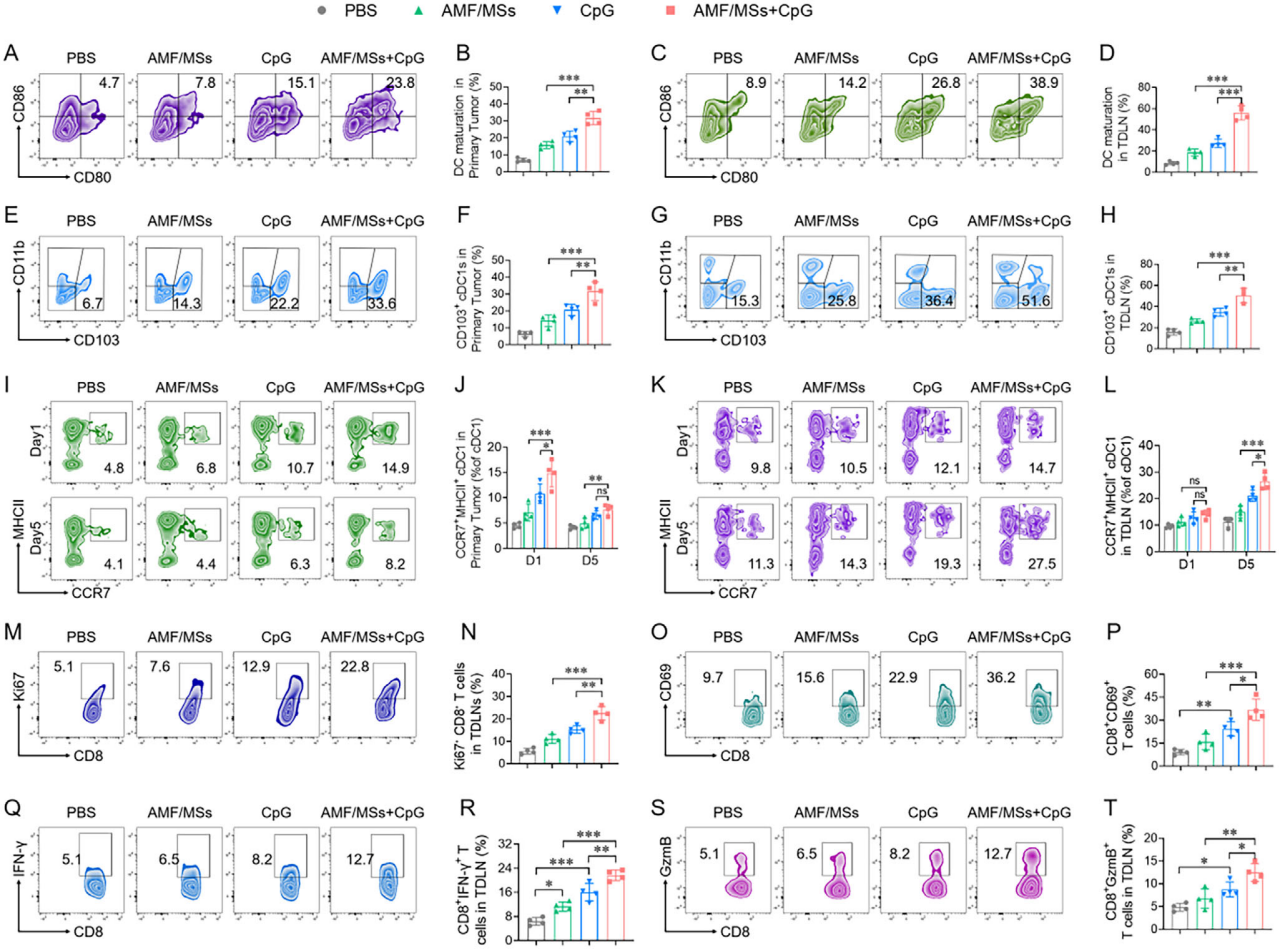
cDC1 Activation across the Tumor–TDLN Axis and Functional CD8^+^ T‐Cell Priming in TDLNs. (A, B) Representative flow cytometry plots and quantification of total DCs in treated primary tumors. (C, D) Representative flow cytometry plots and quantification of total DCs in TDLNs. (E, F) Representative flow cytometry plots and quantification of CD103^+^ cDC1s in treated primary tumors. (G, H) Representative flow cytometry plots and quantification of CD103^+^ cDC1s in TDLNs. (I, J) Representative flow cytometry plots and quantification of CCR7^+^ cDC1s in treated primary tumors at Day 1 and Day 5. (K, L) Representative flow cytometry plots and quantification of CCR7^+^ cDC1s in TDLNs at Day 1 and Day 5. (M, N) Representative flow cytometry plots and quantification of Ki67^+^CD8^+^ T cells in TDLNs. (O, P) Representative flow cytometry plots and quantification of CD69^+^CD8^+^ T cells in TDLNs. (Q, R) Representative flow cytometry plots and quantification of IFN‐γ^+^CD8^+^ T cells in TDLNs. (S, T) Representative flow cytometry plots and quantification of GZMB^+^CD8^+^ T cells in TDLNs. Data are presented as mean ± s.d. (*n* = 4 mice per group). Statistical significance was assessed by one‐way ANOVA with Tukey's multiple comparisons test. ^*^ *p* < 0.05, ^**^ *p* < 0.01, ^***^ *p* < 0.001.

These findings suggest that MTA‐induced tumor disruption and early inflammatory signaling establish a local immune context in which CpG‐mediated innate stimulation more effectively promotes cDC1 enrichment across the treated tumor–TDLN axis.

We further examined CCR7^+^ cDC1s at Day 1 and Day 5 to assess the temporal and compartmental dynamics of cDC1‐associated activation across the tumor–TDLN axis. In treated primary tumors (Figure 7I,J), CCR7^+^ cDC1s increased rapidly at Day 1 and declined by Day 5, particularly after MTA + CpG treatment. In contrast, TDLNs exhibited a delayed increase in CCR7^+^ cDC1s by Day 5 (Figure 7K,L), with the highest level observed in the MTA + CpG group. This reciprocal kinetic pattern suggests early CCR7‐associated cDC1 activation in the ablated TME, followed by delayed enrichment of CCR7^+^ cDC1s in TDLNs. Together with the superior therapeutic efficacy of Day 0/Day 2 CpG administration, these results support temporal coordination of CpG delivery with the early post‐MTA inflammatory window to promote downstream lymph‐node immune priming.

We next evaluated whether this cDC1‐associated response was accompanied by functional CD8^+^ T‐cell priming in TDLNs. MTA + CpG markedly increased Ki67^+^CD8^+^ T cells (Figure 7M,N) and CD69^+^CD8^+^ T cells (Figure 7O,P), indicating enhanced proliferation and early activation. Moreover, IFN‐γ^+^CD8^+^ T cells (Figure 7Q,R) and GZMB^+^CD8^+^ T cells (Figure 7S,T) were most abundant in the MTA + CpG group, suggesting strengthened effector cytokine production and cytotoxic programming. These data indicate that temporally coordinated CpG administration after MTA promotes functional activation of CD8^+^ T cells in TDLNs, rather than merely increasing CD8^+^ T‐cell abundance.

Finally, we assessed systemic CD8^+^ T‐cell phenotypes in the spleen. MTA + CpG increased CD44^hiCD62L^lo effector memory‐like CD8^+^ T‐cell subsets (Figure S15A,B) and CD44^hiCD62L^hi central memory‐like CD8^+^ T‐cell subsets (Figure S15A,C) compared with PBS, MTA alone, and CpG alone groups. These systemic memory‐associated phenotypic changes were consistent with the enhanced TDLN CD8^+^ T‐cell activation observed after combined treatment.

Collectively, these data support a mechanistic model in which temporally coordinated CpG administration after MTA promotes a tumor–TDLN immune‐priming cascade characterized by CD103^+^ cDC1 enrichment and CCR7‐associated cDC1 dynamics, functional CD8^+^ T‐cell priming in TDLNs, and systemic remodeling toward memory‐like CD8^+^ T‐cell phenotypes.

To evaluate the requirement for cDC1s in the immune response induced by MTA + CpG, we compared WT and Batf3^−^/^−^ mice and included an untreated WT group as an immune baseline for flow‐cytometric analysis (Figure 8A). The untreated WT group provided a reference for treatment‐associated immune changes, whereas the comparison between MTA + CpG‐treated WT and Batf3^−^/^−^ mice was used to assess the contribution of Batf3‐dependent cDC1s. The results revealed that the antitumor efficacy of MTA + CpG was markedly attenuated in Batf3^−^/^−^ mice compared with treated WT mice (Figure 8B,C). Flow cytometry analysis indicated that these differences were associated with altered immune activation. Compared with untreated WT mice, MTA + CpG‐treated WT mice showed increased tumor‐infiltrating CD8^+^ T cells and IFN‐γ^+^CD8^+^ T cells, whereas these treatment‐associated increases were markedly attenuated in MTA + CpG‐treated Batf3^−^/^−^ mice (Figure 8D–G). Notably, the results of the ELISA experiments indicated that innate immune responses, such as those involving TNF‐α (Figure 8H) and IL‐6 (Figure 8I), decreased significantly in both groups of mice, among which IFN‐γ (Figure 8J) decreased most notably. H&E staining (Figure 8K,L) confirmed that the antitumor effect of the combination therapy in the Batf3^−^/^−^ mouse group was significantly weaker than that in the wild‐type group, and immunohistochemical staining (IHC) further confirmed these findings using the TUNEL index (Figure 8L). In the immunoaddiction large deficiency group, the apoptosis‐inducing effect of the combination treatment group on distant tumors was significantly weakened. The antitumor killing efficacy was much lower than that in the wild‐type group. These findings indicate that Batf3‐dependent cDC1s are required for the efficient conversion of MTA‐induced local tumor disruption and inflammatory signaling into downstream CD8^+^ T‐cell antitumor immunity. This cDC1 dependence further supports the role of cDC1‐mediated immune priming as a key mechanistic component of the MTA + CpG response and provides a rationale for investigating cDC1‐associated biomarkers in future translational studies.

**FIGURE 8:**
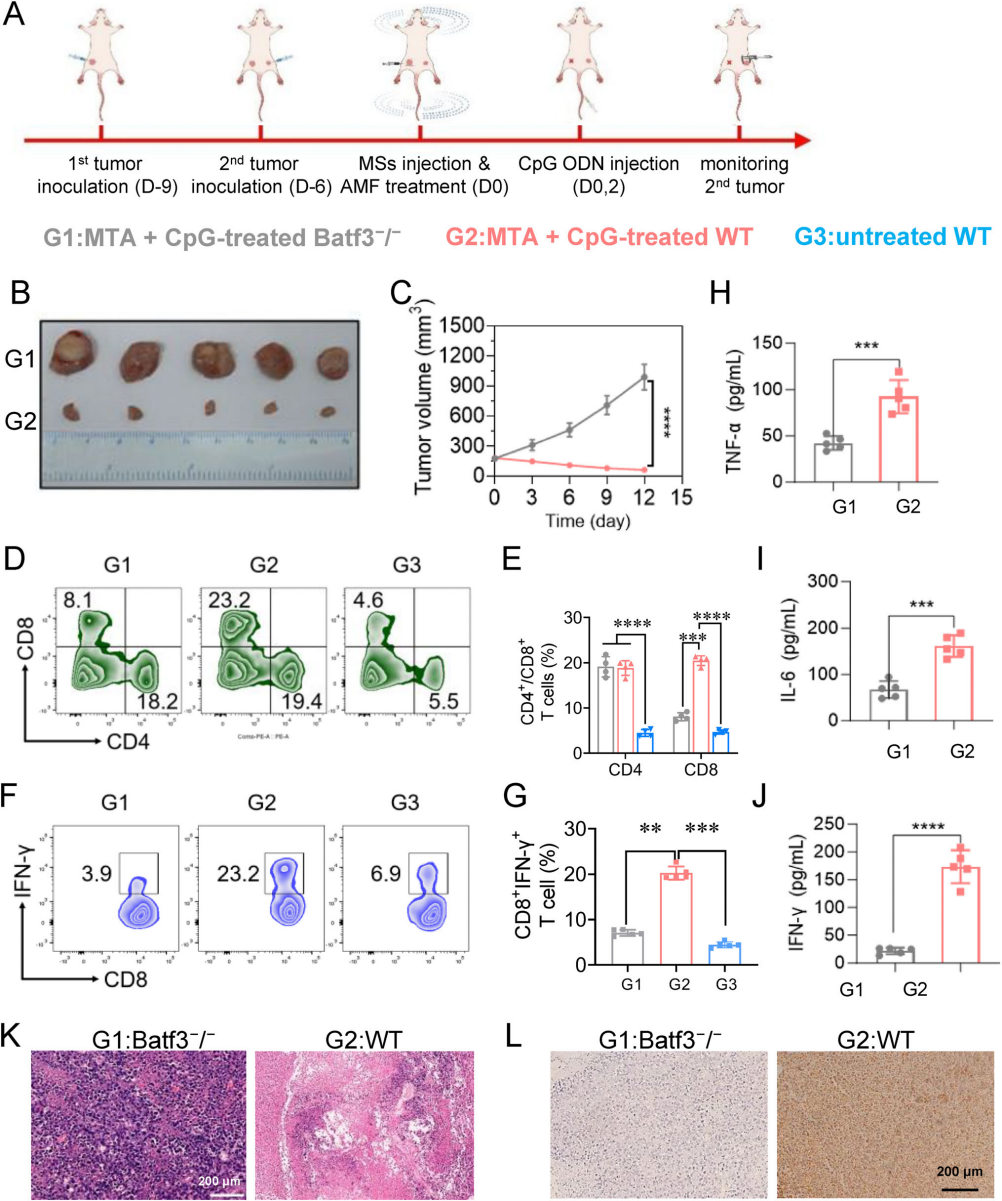
Role of cDC1s in MTA + CpG‐mediated antitumor immunity (Batf3^−^/^−^ model). (A) Experimental schematic of the WT and Batf3^−^/^−^ mouse experiments. (B, C) Digital photographs (B) and growth curves (C) of distal tumors in MTA + CpG‐treated WT and Batf3^−^/^−^ mice (mean ± s.d.; *n* = 5 for photographs and *n* = 7 for growth curves). (D–G) Representative flow‐cytometry plots and quantification of tumor‐infiltrating CD8^+^ T cells (D, E) and IFN‐γ^+^CD8^+^ T cells (F, G) in untreated WT, MTA + CpG‐treated WT, and MTA + CpG‐treated Batf3^−^/^−^ mice (mean ± s.d., *n* = 4). (H–J) ELISA quantification of TNF‐α (H), IL‐6 (I), and IFN‐γ (J) in MTA + CpG‐treated WT and Batf3^−^/^−^ mice (mean ± s.d., *n* = 4). (K, L) H&E (K) and IHC‐TUNEL (L) staining of distal tumor sections from MTA + CpG‐treated WT and Batf3^−^/^−^ mice (scale bar: 200 µm for H&E and 100 µm for TUNEL). Statistical significance for the three‐group flow‐cytometry comparisons in (E, G) was assessed using one‐way ANOVA with Tukey's multiple‐comparisons test, whereas comparisons between MTA + CpG‐treated WT and Batf3^−^/^−^ mice were assessed using Student's *t*‐test. ^*^ *p* < 0.05, ^**^ *p* < 0.01, ^***^ *p* < 0.001, ^****^ *p* < 0.0001.

### 2.5 MTA via Transarterial Embolization for Physical Ablation Validation in Rabbit

Unlike conventional percutaneous thermal ablation, MTA does not require direct insertion of an ablation applicator into the tumor. By delivering the magnetothermal microspheres through the tumor‐feeding artery and subsequently activating them with an external AMF, this strategy may reduce constraints associated with direct percutaneous access to anatomically challenging lesions. By ensuring a homogeneous distribution of magnetic microspheres within the tumor via transarterial embolization (TAE), MTA effectively overcomes the limitations of image‐guided percutaneous ablation, showing significant clinical promise. We conducted preclinical evaluations in large animals.

The MTA efficacy of P@Fe30 MSs was further verified in a rabbit tumor model (Figure 9A). Transarterial embolization was essential for performing MTA with P@Fe30 MSs, enabling precise delivery of the microspheres into the rabbit VX2 tumor‐feeding artery and their accumulation within the tumor. The right femoral artery of the rabbit was punctured with a microcatheter. We performed digital subtraction angiography (DSA) to map the hepatic and tumor‐feeding arteries, followed by superselective catheterization. P@Fe30 MSs were then infused via the microcatheter at a slow rate until vascular stasis was complete (Figure 9B; Figure S16).

**FIGURE 9:**
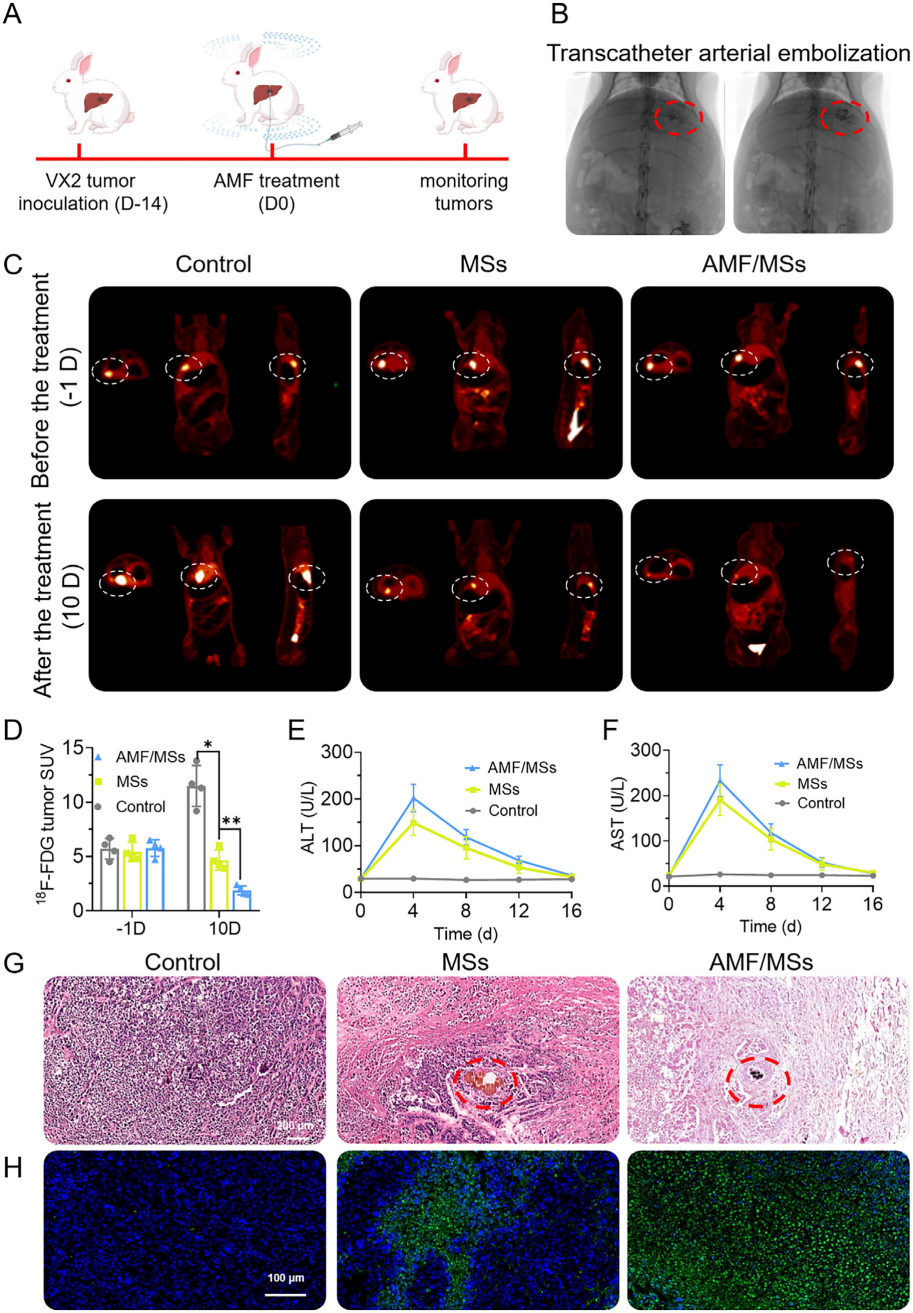
Preclinical Evaluation of MTA via Transarterial Embolization in Rabbit VX2 Liver Tumors. (A, B) DSA images showing catheterization and embolization of P@Fe30 MSs into the VX2 tumor (red dashed line). (C) Representative ^1^ ^8^F‐FDG PET images pre‐ and post‐treatment (tumor outlined in white). (D) Quantitative analysis of Maximum Standardized Uptake Value (SUVmax) (mean ± s.d., *n* = 4 rabbits). (E, F) Serum levels of ALT (E) and AST (F) over time (mean ± s.d., *n* = 4). (G) H&E staining of tumor tissues (red do line indicates microspheres) (scale bar = 200 µm). (H) TUNEL images of tumor tissues (scale bar = 100 µm). Statistical significance was calculated using one‐way ANOVA. ^*^ *p* < 0.05, ^**^ *p* < 0.01.

To visually evaluate the therapeutic effect, PET imaging was performed using ^18^F‐fluorodeoxyglucose (^18^F‐FDG). This provides major advantages in animal studies, including noninvasive, real‐time tracking of therapeutic response with high sensitivity. At baseline, ^18^F‐FDG PET imaging revealed intense radiotracer uptake in the hepatic tumors across all groups, indicating high glycolytic activity. After 10 days of treatment, the metabolic signal expanded in the control group, which was consistent with tumor progression. While microsphere embolization alone (MSs) led to a relative reduction in the metabolically active volume, the most significant suppression was observed in the MTA (AMF/MSs) group, which exhibited only faint ^18^F‐FDG signals, suggesting a potent antitumor effect (Figure 9C). Further statistical analysis of the standardized uptake values (SUVs) confirmed these results (Figure 9D). As a key parameter, the SUVs serve to quantify ^18^F‐FDG uptake, thereby facilitating the evaluation of tumor malignancy and treatment efficacy.

The safety profile of the MTA procedure was confirmed in our study. Transient impairment in liver function was observed in all groups (Figure 9E,F). Transient increases in serum alanine aminotransferase (ALT) and aspartate aminotransferase (AST) levels were observed on Day 4 after treatment, indicating a temporary hepatic effect. Notably, these enzyme levels subsequently normalized and returned to baseline by approximately Day 16.

Histopathological evaluation of tumor tissues corroborated the strong therapeutic effect of MTA. H&E‐stained sections revealed extensive tumor cell necrosis in the MTA group, which was markedly more pronounced than that in all the other groups (Figure 9G). In addition, TUNEL staining (Figure 9H) verified the efficacy of MTA by revealing significant apoptosis in tumor tissues, which underlies the observed suppression of HCC. Crucially, translating high‐temperature magnetic hyperthermia into a safe in vivo ablation modality relies on the intrinsic, physiologically driven safety mechanism of TAE. In liver interventions, thermal safety is largely governed by tissue hemodynamics. Here, the PLA@Fe_3_O_4_ MSs exert a dual function as efficient heat generators and physical embolic agents. The induced blood stasis effectively traps heat within the tumor bed, rapidly inducing coagulative necrosis. Our histological evaluation supports this safety profile at both the local and systemic levels: after embolization followed by MTA, the newly added interface H&E shows that necrosis was predominantly confined to the embolized tumor side with a sharp boundary at the tumor–liver interface (Figure S17), while major non‐target organs exhibited no histopathological evidence of thermal injury (Figure S18). This preservation of normal tissue architecture, further corroborated by the recovery of liver function indicators (ALT/AST), demonstrates that intrinsic thermal confinement ensures procedural safety, thereby mitigating the necessity for complex, invasive real‐time intratumoral thermometry.

While the systemic immune activation induced by temporally coordinated MTA + CpG treatment was robustly demonstrated in the murine bilateral tumor model, the rabbit VX2 study was purposefully designed to evaluate the physical MTA modality and clinical feasibility of the TAE platform in a large organ with human‐like hepatic vascular anatomy. The specific immunological validation in rabbits was limited by the lack of comprehensive antibody panels. Consequently, the potent locoregional ablation efficacy and safety confirmed here pave the way for future translational investigations of the full combinatorial immunotherapy. Collectively, these findings underscore the robust clinical feasibility of engineered magnetothermal microsphere‐mediated ablation, demonstrating considerable translational potential for future medical applications.

## 3 Discussion

In this study, we developed a PLA@Fe_3_O_4_ magnetic microsphere platform for transarterial, remotely activated magnetic thermal ablation of HCC. The SPG membrane emulsification process enabled controllable microsphere fabrication with stable morphology, favorable biocompatibility, and efficient magnetothermal performance. Compared with freely circulating magnetic nanoparticles, microscale embolic agents can be enriched through the arterial route, thereby increasing local magnetic material density and reducing systemic dispersion. Under AMF exposure, P@Fe30 MS‐mediated MTA achieved localized tumor‐cell killing and induced immunogenic cell death‐associated danger signaling, providing a basis for integrating local ablation with immune adjuvant therapy.

A key finding of this work is that CpG administration after MTA is most effective when coordinated with the early post‐ablation immune window. MTA rapidly induced ICD‐associated danger signaling and IFN‐β upregulation, establishing an inflammatory context favorable for subsequent immune priming. Consistently, CpG treatment on Day 0/Day 2 after MTA produced stronger distant untreated tumor suppression and survival benefit than delayed treatment on Day 4/Day 6. These data provide direct experimental support for temporal coordination between MTA‐induced tumor disruption and CpG‐mediated innate immune activation, although broader dosing intervals and tumor models will be required to further define the optimal therapeutic window. Because free CpG was administered intravenously in the present study, off‐target systemic TLR9 stimulation remains an important translational consideration. Future studies should therefore evaluate dose reduction as well as locoregional or controlled‐release CpG delivery strategies to enhance exposure within the ablated tumor–TDLN immune‐priming axis while limiting unnecessary systemic immune activation.

Mechanistically, MTA + CpG promoted a tumor–TDLN immune‐priming cascade. Combined treatment increased total DCs and CD103^+^ cDC1s in treated tumors and TDLNs, while CCR7^+^ cDC1s showed an early increase in treated tumors followed by delayed enrichment in TDLNs. This kinetic pattern supports a cDC1‐associated immune‐priming axis linking the ablated tumor microenvironment with downstream lymph‐node responses. Importantly, this response was accompanied by functional activation of CD8^+^ T cells in TDLNs, as shown by increased Ki67^+^, CD69^+^, IFN‐γ^+^, and GZMB^+^ CD8^+^ T cells. The increase in effector memory‐like and central memory‐like CD8^+^ T‐cell phenotypes in the spleen further suggests systemic memory‐associated immune remodeling. Although these findings support cDC1‐dependent immune priming and systemic memory‐associated CD8^+^ T‐cell remodeling, phenotypic analyses alone cannot establish durable tumor‐protective immune memory. Future antigen‐specific T‐cell analyses and long‐term tumor rechallenge studies will therefore be required to determine the durability, antigen specificity, and protective capacity of the immune response elicited by MTA + CpG.

The functional importance of cDC1s was further supported by the attenuated therapeutic and immune responses in Batf3^−^/^−^ mice. Loss of Batf3‐dependent cDC1 development markedly reduced the antitumor effect of MTA + CpG and impaired CD8^+^ T‐cell activation, indicating that cDC1s are required for the full immune‐priming activity of this regimen. Moreover, the Batf3^−^/^−^ experiment should be interpreted as evidence for the requirement of Batf3‐dependent cDC1s in the overall immune‐priming cascade rather than as identification of a specific cDC1‐dependent antigen‐processing step. Antigen uptake, intracellular processing, CCR7‐associated migration, MHC‐I cross‐presentation, and costimulatory activation are functionally interconnected processes, and the present knockout experiment does not resolve which of these individual steps is selectively responsible for the impaired response. Although our OVA model‐antigen assay independently demonstrates enhanced antigen‐specific MHC‐I presentation by BMDCs following MTA and CpG treatment, this assay does not establish that the Batf3^−^/^−^ phenotype results specifically from defective cross‐presentation. Dedicated step‐resolved studies will therefore be required to define the precise cDC1 function that is indispensable in this therapeutic context. Importantly, this mechanism also distinguishes the present CpG‐based strategy from our previous work combining MTA with anti‐PD‐1 therapy [48]. Whereas checkpoint blockade mainly acts at the effector phase by relieving inhibitory T‐cell signaling, CpG acts upstream by enhancing innate immune activation and dendritic‐cell licensing. The additional benefit observed with MTA + CpG + anti‐PD‐1 suggests that CpG‐mediated immune priming and checkpoint blockade may be mechanistically complementary. From a translational perspective, MTA bridges transarterial embolization and local thermal ablation. Unlike conventional TACE, which combines arterial embolization with intra‐arterial chemotherapy, PLA@Fe_3_O_4_ microspheres function as both embolic agents and magnetothermal transducers, enabling vascular stasis followed by externally triggered thermal ablation without incorporated cytotoxic drugs. MTA also differs from image‐guided percutaneous ablation because heat is generated from artery‐delivered microspheres rather than a directly inserted ablation applicator, potentially reducing constraints associated with percutaneous access to anatomically challenging lesions. In the rabbit VX2 model, AMF‐triggered MTA produced greater metabolic suppression and tumor destruction than microsphere embolization alone, supporting an additional magnetothermal contribution. Together with the ICD‐associated immune effects observed in mice, these findings position MTA as a chemotherapy‐free transarterial ablation platform integrating embolization, remote thermal activation, and immunomodulation compatibility. Although direct comparisons with established TACE and percutaneous ablation modalities will be required to define its relative clinical advantages, the present study establishes the feasibility of integrating transarterial MTA with temporally coordinated CpG immunotherapy to promote cDC1‐dependent systemic antitumor immune responses.

## Author Contributions

Xingwei Sun and Jiakun Yang designed the project. Jiakun Yang, Nailin Yang, Shuangshuang Ji, Wenjun Liang, Siping Meng, and Di Wang performed the material characterization, performance testing, and animal experiments. Xingwei Sun, Xuming Bai, and Yong Jin were responsible for manuscript preparation and review.

## Conflicts of Interest

The authors declare no conflicts of interest.

## Supporting information




**Supporting File**: advs78224‐sup‐0001‐SuppMat.docx.

## Data Availability

Data will be made available on request.
