# Supplementary material for: Magnetic Thermal Ablation Combined with Temporally Coordinated CpG Immunotherapy Promotes cDC1‐Dependent Immune Priming in Hepatocellular Carcinoma
Source: Adv Sci (Weinh). 2026 Oct 22:e78224. Online ahead of print. doi: 10.1002/advs.78224 (PMC13690974; doi:10.1002/advs.78224)
Supplement: Supplementary file 1 [file ADVS-9999-e78224-s001.docx]

Supporting Information

**Magnetic Thermal Ablation Combined with Temporally Coordinated CpG Immunotherapy Promotes cDC1-Dependent Immune Priming in Hepatocellular Carcinoma**

**Experimental section**

**1.Materials**

Oleic acid-modified iron oxide (Fe₃O₄) nanoparticles were obtained from Procell Co., Ltd. Polylactic acid was purchased from Macklin Co., Ltd. CpG ODN was acquired from MedChemExpress Co., Ltd. PD-1 mAb was purchased from Thermo Fisher Technology Co., Ltd. (Shanghai). All chemical reagents were of analytical grade and used without further purification.

Antibodies used for flow cytometry were purchased from Thermo Fisher (USA) and used according to the instructions, including anti-CD45-PE-Cyanine5, anti-CD8a-PE-Cyanine7, anti-CD4-FITC, CD3-eFluor506, anti-FOXP3-PE, anti-CD11c-FITC, anti-CD86-PE and anti-CD80-APC, anti-MHCII-APC-eFluor 780, anti-CD103-eFluor 450, anti-CD11b-Super Bright 600, anti-CCR7-PE, anti-CD69-APC, anti-Ki67-eFluor 450, anti-IFN-γ-PE, anti-GZMB-Alexa Fluor 700, anti-CD44-APC, and anti-CD62L-PE. Anti-CD3 for immunohistochemistry was purchased from Biolegend (USA). Antibodies used for immunofluorescence staining were purchased from Abcam (UK) and used according to the instructions, including anti-CRT, anti-HMGB1, and anti-IFN-β. Assay kits were used according to supplier’s protocol, including MTT Cell Proliferation and Cytotoxicity Assay Kit (MTT, Solarbio), annexin V-APC/7-AAD apoptosis kit (Multi sciences, AT105-100)，and Calcein AM cell viability assay kit (Beyotime). Mouse Tumor Necrosis Factor-α Enzyme-Linked ImmunoSorbent Assay Kit (TNF-α, LianShuo Biological), Mouse Interleukin-6 (IL-6) Enzyme-Linked Immunosorbent Assay Kit，and Mouse Interferon-γ Enzyme-Linked ImmunoSorbent Assay Kit (IFN-γ, LianShuo Biological) were used according to supplier’s protocol.

**2. Experimental Methods**

2.1. Preparation of PLA@Fe_3_O_4_ MSs

PLA@Fe_3_O_4_ MSs with different Fe_3_O_4_ contents were prepared using the SPG film emulsification method. Preprepared nanosized iron (III) oxide, a PEG solution and an SDS aqueous solution of polylactic acid (PLA) were uniformly mixed and added to the SPG membrane emulsification device under temperature control. By adjusting the emulsification pore size (20 microns) and pressure (0.5 mBar), PLA@Fe_3_O_4_ MSs with a diameter of approximately 50 μm were obtained. PLA@Fe_3_O_4_ MSs with different Fe_3_O_4_ contents (X%) are denoted as P@FeX.

2.2. Characterization

Bright-field images of the PLA@Fe_3_O_4_ MSs were obtained using an inverted microscope (Nikon, Ts2R). The corresponding element mapping was accomplished using an SEM instrument equipped with an energy dispersive spectrometer (EDS). ImageJ was used to determine the diameter of each group of microspheres, and distribution maps were drawn on the basis of the percentage of each size. The morphologies of the nanoiron oxide and P@Fe30 MSs were determined by scanning electron microscopy (SEM). NOVA (NanoSEM 230, FEI) was characterized at 200 kV. The stability of P@Fe30 MSs was determined in PBS or 10% fetal bovine serum by 100-fold dilution or stored at 4 °C for 30 days. Unloaded PLA microspheres were incubated in physiological saline at 37 °C for 60 days. At predetermined time points, the microspheres were collected, washed with deionized water, dried to constant weight, and weighed. Residual mass was calculated relative to the initial dry mass, and morphology was examined by optical microscopy (n = 3). P@Fe30 microspheres were incubated in physiological saline at 37 °C for 30 days. At predetermined time points (Days 0, 1, 3, 7, 14, 21, and 30), the incubation medium was collected and analyzed for iron content by ICP-MS after appropriate sample preparation. Cumulative iron release was expressed as a percentage of the initial Fe content in the microspheres. Three independent samples were analyzed at each time point.To assess stability under embolic conditions, P@Fe30 MSs were incubated in fresh rabbit plasma at 37 °C for 48 h, and their size were evaluated. The surface elemental composition of the P@Fe30 MSs was characterized by X-ray photoelectron spectroscopy (XPS, model: Thermo Scientific ESCALAB 250Xi). The magnetic properties of the material were characterized at room temperature (300 K) using a vibrating sample magnetometer (VSM, model: Lake Shore 7404). An alternating magnetic field was generated using a high-frequency induction heating device (TGG-60kw; Guangdong Taiguan Power Supply Technology Co., Ltd.) to evaluate the AMF-induced magnetothermal performance of PLA@Fe_3_O_4_ MSs with different Fe_3_O_4_ contents (10%, 20% and 30%), which was equipped with two heating coils of different radii (5.0 and 15.0 cm). The temperature and thermal images were detected and recorded by an infrared thermal imager (Fotric 225). All thermal imaging photos were obtained by Fotric 225 and analyzed by Analyz IR software.

2.3 Cellular experiments

H22, VX2, and L929 cell lines were purchased from the Chinese Academy of Sciences (Shanghai) Cell Bank. The cell culture dishes and 96-well plates were purchased from Thermo Fisher (Shanghai) Technology Co., Ltd. In the subsequent in vivo and in vitro experiments, AMF/MSs supported the heating P@Fe30 MSs under an alternating magnetic field. CpG ODN 1826 (5′-TCCATGACGTTCCTGACGTT-3′) was purchased from MedChemExpress Co., Ltd. The working concentration of CpG ODN 1826 for the in vitro experiments was 2 μM unless otherwise specified.

In hemolysis studies, red blood cells obtained by processing the blood of healthy mice were dispersed in 0.9% sodium chloride to obtain a 2% (v/v) cell suspension. The samples were incubated at 37 °C for 3 hours after they were mixed with an equal volume of P@Fe30 MSs. Sodium chloride normal saline (0.9%) and ultrapure water (DIW) were used as negative and positive controls, respectively. The hemolysis rate (HR) was calculated using the following formula: HR (%) = (AS - ANC)/(APC - ANC) × 100, where AS, ANC, and APC represent the ultraviolet absorbance of the sample, the negative control and the positive control, respectively.

For in vitro magnetothermal therapy, mouse H22 cells, L929 cells, and VX2 rabbit hepatoma cells were exposed to P@Fe30 MSs with an alternating magnetic field (AMF) for 5 minutes; the therapeutic temperatures of these cells were 42 °C (f_appl_ = 500 kHz, H_appl_ = 4.0 × 10^^3^ A m^−1) and 48 °C (f_appl_ = 500 kHz), respectively. H_appl_ = 5.0 × 10^^3^ A m^−1 and 54 °C (f_appl_ =500 kHz, H_appl_ = 6.0 × 10^^3^ A m^−1).

The effect of MTA on the ultrastructure of tumor cells was investigated using a biological transmission electron microscope (Hitachi HT7800 transmission electron microscope).

To evaluate apoptosis caused by MTA in vitro, the treated cells were stained with an Annexin V-allophycocyanin (APC) and 7-amino-actinomycin D (7-AAD) apoptosis detection kit for 10 minutes. The collected cells were washed with phosphate buffer solution and tested using a flow cytometer.

In terms of the in vitro cell survival rate, the MTT method was used to determine the relative survival rates of cells after different treatments. For in vitro live/dead double staining, the treated cancer cells were stained with Calcein AM (AM, live cells) and propidium iodide (PI, dead cells). All the fluorescence images were obtained by confocal laser scanning microscopy (CLSM, Zeiss Axio-Imager LSM-800).

For the mechanistic in vitro studies, controlled MTA conditions of 42, 48, and 54 °C were used to evaluate temperature-dependent tumor-cell viability, apoptosis, and ICD-associated responses. To additionally assess the cellular consequence of the higher ablative temperature observed during in vivo MTA, H22 cells were exposed to AMF/MS-mediated heating reaching a peak temperature of approximately 81 °C. Following treatment, the cells were stained with Calcein AM and PI and examined by confocal fluorescence microscopy to visualize viable and dead cells. The approximately 81 °C condition was used as a high-temperature ablative assessment rather than for quantitative classification of apoptosis or individual ICD-associated pathways.

To evaluate the surface expression level of CRT in vitro, the treated H22 cells were collected. First, they were fixed with 4% paraformaldehyde for 15 minutes (4 °C), and then blocked at room temperature with 5% BSA for 1 hour. The cells were incubated with anti-CRT antibodies (Abcam, ab2907) at a 1:200 dilution for 2 h, followed by Alexa Fluor 488-labeled secondary antibody (Invitrogen, A-11008, 1:500 dilution) in the dark for 1 h. Finally, the cells were subjected to BD FACS Celesta flow cytometry (BD Biosciences), and the fluorescence intensity was analyzed using FlowJo v10.8 software. The release of HMGB1 was detected using a HMGB1 ELISA Kit (Sigma-Aldrich, RAB0393) in accordance with the associated instructions. The release of IFN-β at 24 h after treatment was detected using an IFN-β ELISA Kit (MultiSciences, EK2236) in accordance with the associated instructions. To quantify extracellular ATP release, culture supernatants were collected from H22 cells after the indicated treatments and extracellular ATP was measured using an ATP assay kit according to the manufacturer’s instructions(Beyotime, S0026).

BMDCs were generated from the bone marrow of female BALB/c mice (6–8 weeks old) in the presence of GM-CSF according to a previously reported protocol. ^[1-2]^. Briefly, bone marrow cells were cultured in RPMI-1640 medium supplemented with 10% FBS, 1% penicillin–streptomycin, and recombinant murine GM-CSF (20 ng mL⁻¹), and BMDCs were collected on Day 7. For the maturation assay, untreated or MTA-treated H22 cells (500 kHz, 6.0 kA m⁻¹, 5 min, approximately 54 °C) were co-cultured with BMDCs at an H22:BMDC ratio of 1:1 for 24 h in the absence or presence of CpG ODN 1826 (2 μM). BMDC maturation was quantified as the percentage of CD80⁺CD86⁺ cells within gated CD11c⁺ BMDCs by flow cytometry. Culture supernatants were collected for TNF-α and IL-6 quantification by ELISA.

To assess antigen-specific MHC-I presentation by BMDCs, B16-OVA cells were untreated or subjected to P@Fe30 MS-mediated MTA (500 kHz, 6.0 kA m⁻¹, 5 min, approximately 54 °C). Following treatment, adherent and non-adherent tumor cells were collected, pooled, washed twice with PBS, and co-cultured with C57BL/6-derived BMDCs at a B16-OVA:BMDC ratio of 1:1 for 24 h, with or without CpG ODN 1826 (2 μM). Cells were then subjected to Fc-receptor blocking with anti-CD16/CD32 (clone 2.4G2), followed by surface staining for CD11c, MHCII, and H-2Kᵇ–SIINFEKL (clone 25-D1.16) under non-permeabilizing conditions. H-2Kᵇ–SIINFEKL expression was quantified as the mean fluorescence intensity (MFI) within gated CD11c⁺MHCII⁺ BMDCs using a BD FACSCelesta flow cytometer. Data were analyzed using FlowJo v10 (n = 4 independent samples).

2.4 In vivo therapeutic effect

The animal experiments used in this study were conducted in accordance with the experimental protocol approved by the Laboratory Animal Center of Soochow University (202408A0618). Female BALB/c mice (4–6 weeks old) and female Batf3^-/-^ mice (BALB/c, 6–8 weeks old) were purchased from Suzhou Jingwei Yu Biotechnology Co., Ltd.

A bilateral H22 subcutaneous tumor model was constructed. A total of 2×10^6^ H22 cells were inoculated into the right hip of each BALB/c mouse as the primary tumor, and 0.5×10^6^ H22 cells were inoculated into the left hip of the same mouse to simulate distant metastatic tumors. When the tumor volume exceeded 200 mm^3^, the tumor-bearing mice were randomly divided into four groups (n=7): (I) the control group; (II) the AMF/MSs (f_appl_ = 500 kHz, Happl = 10.0 kA·m−1) group; (III) the CpG group; and (IV) the AMF/MSs + CpG group. For Group III, CpG adjuvant was intravenous injected (*i.v.*) into the mice. For group II and group IV, P@Fe30 MSs were injected (*i.t.*) into the right tumor, after which magnetic thermal ablation based on P@Fe30 MSs was performed. For group III and group IV, each mouse was given 100 μg of CpG by intravenous injection (*i.v.*) on the 0 and 2 days after magnetic hyperthermia ablation.

For the primary tumor on the right side, the mice in each group were completely resected by magnetic thermal ablation or surgery. For the distant metastatic tumor on the left side, tumor length (L, the longest diameter) and width (W, the perpendicular shorter diameter) were measured every 3 days using calipers, and tumor volume was estimated as **V = L × W²/2**. During AMF exposure, temperature changes were monitored using an infrared thermal imager. After the mice were sacrificed, tumors were stained with hematoxylin and eosin (H&E) and immunohistochemistry.

To determine the contribution of CD8⁺ T cells to the systemic antitumor effect of MTA combined with CpG, an in vivo CD8⁺ T-cell depletion experiment was performed using the bilateral H22 tumor model. Mice were assigned to four groups: PBS control, CD8 depletion control, MTA + CpG, and MTA + CpG + CD8 depletion groups (n = 7 per group). For CD8⁺ T-cell depletion, mice in the CD8 depletion control and MTA + CpG + CD8 depletion groups received anti-mouse CD8β antibody (clone 53-5.8, Thermo Fisher) at 200 μg per mouse by intraperitoneal injection on Days −3, 0, 5, and 10 relative to MTA treatment. MTA was performed on Day 0, and CpG ODN was administered at 100 μg per mouse on Days 0 and 2 as described above. The efficiency of CD8⁺ T-cell depletion was verified by flow cytometric analysis of peripheral blood using CD3 and CD8α staining. Distal tumor dimensions were measured every 3 days, and tumor volumes were calculated as described above.To evaluate the temporal dependence of CpG administration after MTA, bilateral H22 tumor-bearing mice were randomly assigned to four groups: PBS control, AMF/MSs, AMF/MSs + CpG delayed treatment, and AMF/MSs + CpG early treatment. For MTA-containing groups, P@Fe30 microspheres were injected into the primary tumor, followed by AMF-mediated ablation. CpG ODN was administered intravenously at 100 μg per mouse either during the early post-MTA window on Day 0 and Day 2 or during the delayed window on Day 4 and Day 6. Distal tumor growth was monitored every 3 days. Mouse survival was recorded. Distal tumors were collected for H&E and TUNEL staining.

To compare the CpG-containing MTA regimen with checkpoint blockade-based therapy, bilateral H22 tumor-bearing mice were randomly divided into four groups: PBS control, anti-PD-1, AMF/MSs + anti-PD-1, and AMF/MSs + CpG + anti-PD-1.

For MTA-containing groups, P@Fe30 microspheres were injected into the primary tumor followed by AMF-mediated ablation. CpG ODN was administered intravenously at 100 μg per mouse on Day 0 and Day 2 after MTA. Anti-PD-1 antibody was administered intraperitoneally at 200μg per mouse on Days 1,3,5. Distal tumor growth and mouse survival were monitored. Distal tumors were collected for H&E and TUNEL staining.

The Batf3^-/-^ female mice (BALB/c) aged 6–8 weeks used in this study were obtained from Suzhou Jingwei Biology Co., Ltd. The mice were divided into WT Combination and Batf3^-/-^ Combination groups (cDC1 deficient), with seven mice per group. The WT combination group consisted of female Balb/c mice (4–6 weeks old), whereas the Batf3^-/-^ combination group consisted of female Batf3^-/-^ mice to simulate the absence of cDC1. Both groups received combined treatment with AMF/MSs + CpG (100 μg), and the treatment methods and flow cytometry immune assessment steps were consistent with those described earlier.

2.5 In vivo immune evaluation

To characterize immune-cell populations in distant tumors, tumors were collected from mice in the indicated treatment groups at the specified time points (n = 4 per group unless otherwise indicated). Tumor tissues were minced into small pieces, mechanically dissociated, and treated with 0.25% trypsin-EDTA at 37 °C for 5 min. The resulting cell suspensions were filtered through a 70 μm nylon mesh and washed with PBS. Single-cell suspensions from treated primary tumors, distant tumors, tumor-draining lymph nodes (TDLNs), spleens, and peripheral blood were prepared for flow-cytometric analysis as indicated.

Before antibody staining, cells were incubated with anti-mouse CD16/CD32 for Fc-receptor blocking. Surface staining was performed using the indicated fluorophore-conjugated antibodies for 30 min at 4 °C in the dark.

For intracellular IFN-γ analysis, single-cell suspensions were stimulated for 5 h at 37 °C with a cell-stimulation cocktail containing PMA and ionomycin in the presence of brefeldin A and monensin. Cells were subsequently stained for the indicated surface markers, fixed and permeabilized, and stained intracellularly for IFN-γ. For intracellular GZMB, Ki67, and FoxP3 detection, no ex vivo restimulation was performed. Following surface staining, cells were fixed and permeabilized using the Foxp3/Transcription Factor Staining Buffer Set and subsequently stained with the corresponding intracellular antibodies according to the manufacturer’s instructions.

For flow-cytometric analysis, debris-like events were minimized on the basis of forward- and side-scatter characteristics, and doublets were excluded using FSC-A/FSC-H gating. No dedicated fixable Live/Dead viability dye was included in the original in vivo immune-profiling panels; therefore, scatter-based gating was used to minimize debris but was not considered equivalent to specific exclusion of nonviable cells.

For tumor and TDLN dendritic-cell analysis, gated CD45⁺ leukocytes were further analyzed for CD11c, MHCII, CD103, CD11b, and CCR7 expression as indicated. T-cell populations were identified from gated CD45⁺ cells using CD3, CD4, and/or CD8α, followed by analysis of CD69, Ki67, IFN-γ, GZMB, FoxP3, CD44, or CD62L as appropriate.

Compensation was performed using single-stained controls, and fluorescence-minus-one controls were used to define positive gates where appropriate. Samples were acquired using a BD FACSCelesta flow cytometer (BD Biosciences), and data were analyzed using FlowJo v10. Representative sequential gating strategies for the immune-cell populations analyzed in distant tumors, treated primary tumors, TDLNs, and spleens are provided in Figure S12.

In addition, immunohistochemical experiments were performed using a TUNEL assay kit in accordance with the instructions in the manual. The antibodies used for flow cytometry are listed in the Materials section and were used according to the manufacturers’ instructions.

To evaluate early immune-associated signals after MTA, primary tumors were collected 24 h after AMF/MSs-mediated ablation. Tumor tissues were fixed in 4% paraformaldehyde, embedded in paraffin or OCT, and sectioned. After antigen retrieval when necessary, sections were blocked with 5% BSA and incubated with primary antibodies against CRT, HMGB1, or IFN-β at 4 °C overnight. After washing, sections were incubated with fluorophore-conjugated secondary antibodies for 1 h at room temperature. Nuclei were counterstained with DAPI. Fluorescence images were acquired using a confocal microscope. Fluorescence intensity was quantified using ImageJ software when applicable.

For absolute immune-cell quantification, the total number of cells recovered from each individual tissue sample after dissociation was recorded before flow-cytometric staining. Absolute numbers of individual immune-cell populations were reconstructed from the recorded total cell yield and the corresponding cumulative frequency obtained through the sequential flow-cytometric gating hierarchy.For distant untreated tumors, the absolute numbers of CD45⁺ leukocytes and CD8⁺ T cells were normalized to the mass of the tumor tissue used for dissociation and expressed as cells mg⁻¹ tumor. For treated primary tumors, absolute CD103⁺ cDC1 numbers were similarly normalized to the mass of the tissue used for flow-cytometric analysis and expressed as cells mg⁻¹ tumor. For TDLNs, absolute CD103⁺ cDC1 numbers were expressed as cells per TDLN sample. The same gating definitions used for the corresponding relative-frequency analyses were applied for absolute quantification.

Absolute cell number was calculated as:

**Absolute subset number = total recovered cell number × cumulative gated fraction of the corresponding subset**

For tumor samples:

**Cell density (cells mg⁻¹ tumor) = absolute subset number / tissue mass used for dissociation (mg)**

All absolute cell numbers were calculated individually for each animal before group-level statistical analysis.

2.6 In vivo magnetothermal ablation

Ultrasound-guided VX2 tumor tissue was implanted into the left lobe of the liver of New Zealand white rabbits, and tumor formation was confirmed by CT scan approximately 12 days later. Rabbits with VX2 tumors were subsequently randomly divided into the following three groups (n=4): (I) the control group, (II) the MSs group (1 ml), and (III) the AMF/MSs group (f_appl_ = 500 kHz, Happl = 10.0 kA·m^−1^). The femoral artery was punctured to enter the microcatheter. Under the guidance of DSA, the microcatheter could superselectively enter the left hepatic artery. Angiography showed the blood supply of the tumor. One milliliter of P@Fe30 MSs was embolized to the tumor site. Liver function indicators were tested every four days. One week after the treatment, 18F-deoxyglucose positron emission tomography (PET) imaging was performed to evaluate the biological activity of the tumors in the three groups. Finally, rabbits bearing VX2 tumors were euthanized, and tumor tissue samples were collected for further histopathological (H&E staining) and TUNEL staining evaluations.

2.7 Statistics

All the quantitative experiments were performed in triplicate unless otherwise indicated. The data are presented as the mean ± SD. One-way analysis of variance (ANOVA) was used to analyze the significant differences among groups. Statistical differences in survival were measured by the log-rank test. Significance is expressed as n. s: P > 0.05, ∗p < 0.05, ∗∗p < 0.01, ∗∗∗p < 0.001, and * * * * p < 0.0001.

**3. Supporting Figures**

Figure S1. Representative optical microscopy image of P@Fe40 microspheres prepared under the same SPG processing conditions used for P@Fe30. Increasing the Fe₃O₄ fraction to 40% resulted in markedly less uniform particle formation, with irregular, deformed, and fragmented structures observed. Scale bar: 100 μm.

**Figure S2.** **Stability evaluation of P@Fe30 MSs in rabbit plasma.** Mean diameter were measured at 0 h and 48 h. Data are presented as mean ± s.d. (n = 3).

**Figure S3.** **In vitro degradation of unloaded PLA microspheres.** (A) Residual mass of PLA microspheres during incubation in physiological saline at 37 °C for 60 days. Data are presented as mean ± s.d. (n = 3 independent samples). (B) Representative optical microscopy images showing morphological changes of PLA microspheres at the indicated time points. Scale bar: 50 μm.

**Figure S4. In vitro iron release from P@Fe30 microspheres.** Cumulative iron release from P@Fe30 microspheres during incubation in physiological saline at 37 °C for 30 days, quantified by ICP-MS. Data are presented as mean ± s.d. (n = 3 independent samples).

**Figure S5. Effects of magnetic microspheres or AMF exposure alone on H22-cell apoptosis.** Representative Annexin V-APC/7-AAD flow cytometry plots and quantitative analysis of H22-cell apoptosis in the PBS, MS-only, and three AMF-only groups. The three AMF-only groups were exposed to the same AMF frequency and duration and to the respective field strengths used for the AMF/MS treatments reaching 42, 48, and 54 °C. Data are presented as mean ± s.d. (n = 3). Statistical significance was assessed by one-way ANOVA with Tukey’s multiple-comparisons test. ns, not significant.

**Figure S6. Live/dead staining of H22 cells following high-temperature MTA.** Representative Calcein AM/PI fluorescence images of H22 cells in the PBS control and AMF/MS-mediated MTA groups. MTA treatment reached a peak temperature of approximately 81 °C. Calcein AM (green) indicates viable cells, whereas PI (red) indicates dead cells. Scale bar = 100 μm.

**Figure S7. In vitro IFN-β production following MTA.** IFN-β levels in the culture supernatant of H22 cells following the indicated treatments were quantified by ELISA at 24 h after treatment. Data are presented as mean ± s.d. (n = 3).

**Figure S8. MTA and CpG enhance antigen-specific MHC-I presentation by BMDCs.** Untreated or MTA-treated B16-OVA cells were co-cultured with C57BL/6-derived BMDCs for 24 h in the absence or presence of CpG. The four groups were B16-OVA, MTA-treated B16-OVA, B16-OVA + CpG, and MTA-treated B16-OVA + CpG. Surface SIINFEKL–H-2Kᵇ complexes on gated BMDCs were detected using the 25-D1.16 antibody and quantified by mean fluorescence intensity (MFI). Data are presented as mean ± s.d. (n = 4 independent samples). Statistical significance was assessed by one-way ANOVA with Tukey's multiple comparisons test. *p < 0.05, **p < 0.01, ***p < 0.001.

**Figure S9. Effect of CpG ODN 1826 on H22 cell viability.** H22 cells were treated with the indicated concentrations of CpG ODN 1826, and cell viability was assessed by MTT assay. Data are presented as mean ± s.d. (n = 5).

**Figure S10.** H&E staining images of major organs of mice in different treatment groups. Scale bar: 200 μm.

**Figure S11. Absolute quantification of key immune-cell populations in the corresponding immune compartments.** (A) Absolute density of total CD45⁺ leukocytes in distant untreated tumors. (B) Absolute density of CD8⁺ T cells in distant untreated tumors. (C) Absolute density of CD103⁺ cDC1s in treated primary tumors. (D) Absolute number of CD103⁺ cDC1s in tumor-draining lymph nodes (TDLNs). Absolute cell numbers were calculated from the recorded total cell yield of each individual sample and the corresponding cumulative flow-cytometric gating frequency. Tumor-associated cell numbers were normalized to the mass of tissue used for dissociation and are presented as cells mg⁻¹ tumor, whereas TDLN cDC1 numbers are presented as cells per TDLN. Data are presented as mean ± s.d. (n = 4). Statistical significance was assessed by one-way ANOVA with Tukey’s multiple-comparisons test. *p < 0.05, **p < 0.01, ***p < 0.001, ****p < 0.0001.

**Figure S12.** Representative flow-cytometric gating strategies used for immune-cell analysis.

**Figure S13. CpG Further Enhances the Systemic Antitumor Effect of MTA Combined with anti-PD-1 Therapy.** (A) Schematic of the bilateral tumor model and treatment regimen. Magnetic microspheres were injected into the primary tumor followed by AMF-mediated MTA. CpG was administered on Day 0/Day 2, and anti-PD-1 was given according to the indicated treatment schedule. (B) Tumor growth curves of distal tumors in different treatment groups (n=5 mice). G1: Control; G2: anti-PD-1; G3: AMF/MSs + anti-PD-1; G4: AMF/MSs + CpG + anti-PD-1. (C) Kaplan-Meier survival curves of mice(mean ± s.d., n=5 mice). (D) H&E staining of distal tumor sections from different treatment groups. (E) IHC-TUNEL staining of distal tumor sections. Scale bar: 200 μm for H&E and 100 μm for TUNEL. Data are shown as mean ± s.d. Statistical significance was calculated using one-way ANOVA for tumor volume and log-rank test for survival analysis. *p < 0.05, **p < 0.01, ***p < 0.001.

**Figure S14. CD8⁺ T-cell depletion attenuates the systemic antitumor effect of MTA combined with CpG.**(A) Schematic illustration of the bilateral H22 tumor model and CD8⁺ T-cell depletion schedule. Anti-CD8 antibody was administered according to the indicated schedule in mice receiving MTA + CpG treatment. (B) Representative flow cytometry plots and quantitative analysis confirming depletion of CD8⁺ T cells in peripheral blood. (C) Growth curves of untreated distal tumors in the PBS, MTA + CpG, and MTA + CpG + CD8 depletion groups (n = 7) Data are presented as mean ± s.d. Statistical significance was assessed by one-way ANOVA with Tukey's post hoc test for tumor volume comparisons. *p < 0.05, ****p < 0.0001.

**Figure S15. Systemic Memory-Associated CD8⁺ T-Cell Phenotypes in the Spleen.** (A–C) Representative flow cytometry plots (A) and quantification of CD44hiCD62Llo effector memory-like (B) and CD44hiCD62Lhi central memory-like (C) CD8⁺ T-cell subsets in the spleen. Data are presented as mean ± s.d. (n = 4 mice per group). Statistical significance was assessed by one-way ANOVA with Tukey's multiple comparisons test. *p < 0.05, **p < 0.01, ***p < 0.001.

**Figure S16.** Embolization Methodology. (A) Percutaneous access establishment; (B) Catheter insertion and positioning ; (C) Representative in vivo computed tomography (CT) images demonstrating transarterial embolization of rabbit hepatic tumor using PLA@Fe₃O₄ magnetic microspheres.

**Figure S17.** H&E staining of the tumor–normal liver interface in the rabbit VX2 liver tumor model after transarterial embolization (TAE) with P@Fe30 microspheres followed by AMF-triggered magnetothermal ablation. A sharp boundary is observed between the necrotic tumor region and the adjacent preserved normal hepatic parenchyma. Scale bar: 100 μm.

**Figure S18.** H&E staining images of major organs of rabbits in different treatment groups. Scale bar: 200 μm.

[1]Yang N, Sun X, Zhou Y, et al. Liquid metal microspheres with an eddy-thermal effect for magnetic hyperthermia-enhanced cancer embolization-immunotherapy[J]. Science Bulletin, 2023, 68(16): 1772-1783.

[2]Lutz M B, Kukutsch N, Ogilvie A L J, et al. An advanced culture method for generating large quantities of highly pure dendritic cells from mouse bone marrow[J]. Journal of immunological methods, 1999, 223(1): 77-92.
